# Loss of tuberous sclerosis complex 2 confers inflammation via dysregulation of nuclear factor kappa-light-chain-enhancer of activated B cells

**DOI:** 10.1186/s12950-025-00464-8

**Published:** 2025-09-26

**Authors:** Darius K. McPhail, Mohammad A.M. Alzahrani, Katie R. Martin, Brian L. Calver, Adrian J. Harwood, Jeffrey P. MacKeigan, David M. Davies, Andrew R. Tee

**Affiliations:** 1 Division of Cancer and Genetics, Cardiff University, Heath Park, Cardiff, CF14 4XN UK; 2 Pediatrics and Human Development, College of Human Medicine, Michigan State University, 400 Monroe Ave, Grand Rapids, MI 49503 USA; 3 Neuroscience and Mental Health Research Institute, Cardiff University, Hadyn Ellis Building, Cathays, Cardiff, CF24 4HQ UK; 4 College of Human Medicine, Michigan State University, 220 Trowbridge Road, East Lansing, MI 48824 USA; 5 Department of Oncology, South West Wales Cancer Centre, Singleton Hospital, Swansea, SA2 8QA UK

**Keywords:** TSC, MTOR, NF-κB, STAT3, IL-6, rapamycin, inflammation

## Abstract

**Background:**

Aberrant activation of mTORC1 is clearly defined in TSC and causes uncontrolled cell growth. While mTORC1 inhibitors show efficacy in stabilising tumour growth in patients with TSC, they are not fully curative. Disease facets of TSC that are not restored with mTOR inhibitors might involve NF-κB. This study aimed to characterise NF-κB in the context of TSC.

**Results:**

Enrichment of NF-κB-regulated genes was observed in TSC patient tumours, SEN/SEGAs, cortical tubers, and a TSC tumour-derived cell line (621 − 101). Highlighting an inflammatory component of TSC, TSC cell models exhibit elevated NF-κB and STAT3 activation. Herein, we report a dysregulated inflammatory phenotype in *TSC2*-deficient cells in which NF-κB promotes autocrine signalling involving IL-6. Notably, mTORC1 inhibition does not block this inflammatory signal to promote STAT3, while NF-κB inhibition is much more effective. Combined mTORC1 and NF-κB inhibition potently prevented the anchorage-independent growth of *TSC2*-deficient cells, whereas mTORC1 inhibition alone was insufficient to prevent colony regrowth after cessation of treatment.

**Conclusion:**

This study reveals autocrine signalling crosstalk between NF-κB and STAT3 in TSC cell models. Furthermore, the data presented indicate that NF-κB pathway inhibitors could be a viable adjunct therapy to the currently available mTORC1 inhibitors for treating TSC.

**Supplementary Information:**

The online version contains supplementary material available at 10.1186/s12950-025-00464-8.

## Background

Tuberous Sclerosis Complex (TSC) is a rare, autosomal dominant genetic condition caused by inactivating mutations in either the *TSC1* or *TSC2* gene. TSC patients are predisposed to kidney, skin, brain, and heart tumours (reviewed in [1]). Renal angiomyolipomas (AMLs) are highly vascularised benign tumours containing both smooth muscle and adipose tissue that occur in ~ 80% of adult TSC patients and are the primary cause of mortality after the age of 30 [2]. TSC-associated brain lesions include subependymal nodules (SENs) and subependymal giant cell astrocytomas (SEGAs), which can result in hydrocephalus [3]. Additionally, TSC patients often present with cortical tubers, which are believed to be epileptic foci in the majority of TSC patients [4]. White matter abnormalities are also common in TSC patients (up to 95%) and likely contribute to the onset, frequency, and severity of seizures [5]. Approximately 90% of TSC patients will experience a seizure within their lifetime. Seizures can be refractory to standard antiepileptic medications, making seizures difficult to treat in approximately one-third of TSC patients [6]. Furthermore, 50% of TSC patients will have some degree of intellectual disability [7].

Currently, one key feature of TSC1/TSC2 biology is well understood: the ability of the TSC1/2 tumour suppressor complex to inhibit growth signalling through mechanistic target of rapamycin complex 1 (mTORC1). The small G protein Rheb, which directly activates mTORC1 kinase activity when GTP-bound, is negatively regulated by the GTPase activating protein (GAP) domain of TSC2 [8]. Consequently, inactivating mutations within either TSC1 or TSC2 favour GTP loading of Rheb and aberrant protein kinase activity of mTORC1, leading to uncontrolled cell growth. mTORC1 inhibitors are now used worldwide to treat TSC patients. Long-term treatment with mTORC1 inhibitors (> 3 years) in TSC patients markedly improved seizures refractory to conventional antiepileptic drugs [9]. Tumour volumes are also reduced by mTORC1 inhibitors, with both angiomyolipomas and SEGAs being reduced by > 60%. While mTORC1 inhibitors alleviate many disease traits of TSC, they do not restore disease to normal (reviewed in [10]). For instance, tumours do not regress completely and grow back when therapy stops. A greater understanding of how the loss of either *TSC1* or *TSC2* can drive disease is needed before additional curative therapies can be developed for TSC.

In this study, we examined differentially expressed genes (DEGs) in TSC patient tumours that highlighted gene sets involved in oxidative stress and inflammation. Oxidative stress is known to activate redox-sensitive transcription factors, such as nuclear factor kappa B (NF-κB). NF-κB is involved in the survival, growth, and migration of cancer cells (reviewed in [11]) and is stimulated by a variety of growth factors and cytokines (reviewed in [12]). Briefly, the NF-κB subunits RelA (p65) and RelB are expressed ubiquitously and reside in their inactive forms in the cytoplasm. The RelA and RelB subunits possess transcriptional activation domains. To activate NF-κB, NF-κB inhibitor alpha (commonly referred to as IκBα) is phosphorylated and inactivated by the IκB kinase (IKK) complex. This causes dissociation, ubiquitination, and subsequent degradation of IκBα. IKK also phosphorylates RelA at S536, promoting the transcriptional activity of NF-κB via the association of homo or heterodimers of NF-κB subunits, most commonly RelA/NF-κB1. These activator inputs unmask the nuclear localisation signals within NF-κB subunits, leading to their nuclear translocation and transcription of proinflammatory NF-κB genes.

STAT3 is a proinflammatory transcription factor that promotes oncogenesis by enhancing tumour survival, motility, and cell proliferation [13]. Phosphorylation of Y705 is the most well-known mechanism of STAT3 activation and typically occurs downstream of cytokine stimulation. For example, IL-6 stimulation results in the phosphorylation of Y705-STAT3. This leads to STAT3 dimerisation and subsequent translocation to the nucleus, where STAT3 homodimers promote proinflammatory gene activation [14]. STAT3 possesses a phosphorylation site on S727, although the functional role of this site is poorly understood. S727 phosphorylation is believed to negatively regulate Y705 STAT3 phosphorylation, thus reducing STAT3 inflammatory activity [15]. However, other studies have reported that S727 phosphorylation is required (alongside Y705 phosphorylation) for maximal STAT3 activation (and tumourigenic signalling) [16].

While mTORC1 inhibitors demonstrate significant clinical applicability, their effectiveness is often limited, and mTORC1 inhibitors are relatively ineffective at reducing the expression of various disease-associated signalling pathways, such as the NF-κB, STAT3, and HIF-1α pathways [17]. For this reason, investigating inflammatory pathways may lead to alternative treatment options for TSC.

Current evidence of NF-κB signalling in TSC is limited and suggests varied dysregulation of NF-κB signalling. One study reported a context-dependent role of TSC2 in NF-κB activity [18]. Small interfering RNA (siRNA)-mediated knockdown of TSC2 was found to increase the activity of NF-κB; however, this effect was observed only in cells with nonfunctional PTEN. It was believed that this occurred downstream of mTORC1. Conversely, the same study reported that when PTEN was restored, *TSC2* knockdown resulted in a decrease in NF-κB activation. This highlights the context-dependent role of TSC2 in the regulation of NF-κB. Another study revealed that mTORC1 inhibition impacted NF-κB activation in *TSC2*-deficient immune cells [19]. Notably, in *TSC2*-deficient cells, the transactivation domain of RelA was inactivated by mTORC1-dependent phosphorylation, resulting in reduced NF-κB activity. Inhibition of mTORC1 reversed the reduction in NF-κB activity and resulted in hyperactivation of NF-κB. Given the possible complex role of dysfunctional NF-κB activity in the pathophysiology of TSC, herein, we sought to further elucidate the role of NF-κB in the context of TSC.

## Methods

### Cell culture and drug treatments

621 − 101 *TSC2*-deficient (*TSC2*−) cells were derived from the renal AML of a TSC patient and possess a homozygous missense mutation in *TSC2* (G1832A), resulting in an R611Q amino acid substitution [20]. Wild-type human *TSC2* was re-expressed to generate 621 − 103 (*TSC2*+) AML cells [21]. *Tsc2*(−/−) and *Tsc2*(+/+) mouse embryonic fibroblasts (MEFs) are immortalised with *Tp53*(−/−), which was originally derived from early-stage embryos in an interbreeding study [22]. Eker rat leiomyoma-derived *Tsc2*-deficient cells (ELT3-V3) and matched control cells reexpressing *Tsc2* (ELT3-T3) were generated by Astrinidis et al. [23] and was a gift from C. Walker (M.D. Anderson Cancer Center, Houston, USA). Cell lines were maintained at 37 °C and 5% CO_2_ in a humidified incubator. Cells were cultured in DMEM (Gibco™, Thermo Fisher Scientific, Oxford, UK) on Techno Plastic Products™-coated tissue culture plasticware (Helena Biosciences Europe, Gateshead, UK) supplemented with foetal bovine serum (FBS) at either 10% (*v*/*v*) or 15% (*v*/*v*) for MEF and ELT3 cells or AML cells, respectively, with 50 IU/mL penicillin and streptomycin. 100 µM rapamycin and 20 mM C188-9 (Merck Life Science UK Ltd, Gillingham, UK) and 20 mM BMS345541 (Selleck Chemicals GmbH, Munich, Germany) drug stocks were made up in dimethylsulfoxide (DMSO) and stored as single-use aliquots at −80 °C. Drugs were added to the culture media at a consistent % (*v*/*v*) DMSO per condition without exceeding 0.5% (*v*/*v*) DMSO. Tumour necrosis factor (TNF) and interleukin 6 (IL-6) (purchased from Abcam, Cambridge, UK) were resuspended in ddH_2_O containing 0.2% (*w*/*v*) bovine serum albumin (BSA) to 100 µg/mL and 50 µg/mL, respectively (stored as single-use aliquots at −80 °C). Cell passage was kept < 30 in AMLs and ELT3 and < 45 in MEFs. Mycoplasma-free frozen cell stocks were used; all cells were routinely checked with a Venor GeM advance mycoplasma detection kit (Minerva Biolabs, Berlin, Germany) according to the manufacturer’s guidelines and were negative for mycoplasma spp.

### Western blotting

Cells were seeded on 60 mm plates and grown to 70–80% confluency prior to treatment. To generate whole cell lysates, cells were washed in ice-cold phosphate-buffered saline (PBS) before direct lysis in sample buffer (62.5 mM Tris-HCl (pH 7.6), 10% (*v*/*v*) glycerol, 2% (*w*/*v*) SDS, and 50 mM fresh dithiothreitol. Samples were sonicated before boiling for 10 min at 95 °C. The samples were then centrifuged at 17,000 × g for 10 min. Protein concentration was determined at OD_660_ using Pierce™ reagent supplemented with ionic detergent compatibility reagent (Thermo Fisher Scientific, Oxford, UK). Protein was separated by denaturing polyacrylamide gel electrophoresis using gradient Invitrogen NuPage™ protein gels (Thermo Fisher Scientific, Oxford, UK). Resolved proteins were subsequently transferred to Immobilon^®^-P polyvinylidene difluoride membranes (Merck Life Science, Dorset, UK). Western blotting was carried out as directed by the antibody manufacturer’s protocols: primary antibodies (Cell Signaling Technology Danvers, USA) and horseradish peroxidase-conjugated secondary antibodies (Merck Life Science, Dorset, UK). Protein bands were detected by enhanced chemiluminescence using Cytiva Amersham™ ECL Select™ western blotting detection reagent (Cytiva, Buckinghamshire, UK).

### Soft agar colony formation assay

BD DIFCO™ Noble Agar (BD Biosciences, Wokingham, Berkshire, UK) was melted in PBS to 1.2% (*w*/*v*) and then diluted in DMEM to yield 0.6% (*w*/*v*) agar. Two milliliters of this solution was added to 6-well plates and left at room temperature for solidification. In each well, 0.3% (*w*/*v*) agar DMEM solution containing 20,000 cells was overlaid on top of the 0.6% (*w*/*v*) agar bottom layer. After setting, media containing the relevant drugs was added, and cell colonies were grown for 2–4 weeks, with the media changed every 72 h to refresh the drugs. Images were taken on an EVOS XL Core camera and analysed in ImageJ. (v1.53) to determine colony diameters. After drug treatment, the media was changed and replaced every 72 h in the absence of drugs for an additional 3 weeks, after which further images were taken.

### RNA sequencing

Cells were washed in ice-cold PBS and lysed in RNAprotect^®^ Cell Reagent (Qiagen, West Sussex, UK). RNA was extracted using QIAshredder^®^ and RNeasy^®^ Mini kits (Qiagen, West Sussex, UK) and was stored at −80 °C. RNA library preparation and sequencing were performed through a commercial service/collaboration with Wales Gene Park (Cardiff University, UK), as described previously [24], except for the Illumina^®^ TruSeq^®^ RNA sample preparation v2 kit (Illumina, Inc., Great Abington, Cambridgeshire, UK), which was used for library preparation according to the manufacturer’s instructions. Following validation, the libraries were normalised to 8 nM, and the pool was sequenced on the MiSeq platform with a 150 cycle, version 3, cartridge (both from Illumina, Inc.) according to the manufacturer’s instructions. Differentially expressed transcripts were identified using the DeSeq2 package in R [25]. All pairwise comparisons in the dataset were analysed. *P* values were corrected for multiple testing using the Benjamini–Hochberg false discovery rate (FDR) method. Bioinformatic work was initially carried out by Wales Gene Park.

### Patient-derived TSC transcriptomic analysis and gene ontology analysis

Samples of TSC patient-derived tumours (*n* = 15) were collected by Prof. J. MacKeigan (Michigan State University, Grand Rapids, MI, USA). Gene expression analysis was performed as previously described [26]. Differentially expressed gene (DEG) analysis was performed with GeneAnalytics (LifeMap Sciences, Inc., Covina, CA, USA). A similar analysis was performed with TSC patient-derived cortical tubers (*n* = 15). Gene Ontology analysis was used to identify dysregulated inflammatory and immune system processes in TSC patient-derived tumours. Datasets were imported into Microsoft Excel to generate volcano plots.

### Transcriptional activation ELISAs

Cells were seeded on 6 cm plates and grown over two days until they reached 80–90% confluence. The media was replaced with serum-depleted media, which included pathway inhibitors or DMSO, where applicable, for 24 h. When assaying cytokine induction, the media was supplemented with TNF or IL-6 for the final 2 h (TNF) or 1 h (IL-6) of treatment, respectively. When the effect of media conditioned by *TSC2*-deficient cells on wild-type cells was assayed, *TSC2*-deficient MEFs or AML cells were grown until 80% confluence, before the media was replaced with serum-free media. The cells were incubated under starvation conditions for 24 h before the conditioned media were collected, briefly centrifuged, and subsequently added to the wild-type cells. For transcription assays, cell lysates were prepared and assayed using a TransAM^®^ STAT3 Transcription Factor ELISA Kit (Active Motif, Waterloo, Belgium) with nuclear preparations following the manufacturer’s instructions.

### Conditioned media ELISAs

Secreted IL-6 and VEGF-A concentrations in the media were measured using R&D Systems Duoset ELISAs and ancillary reagent kits (Bio-Techne Ltd., Abingdon, UK) according to the manufacturer’s instructions. Cells were grown in 12-well plates to 90% confluence. The serum-supplemented media was replaced with serum-supplemented media containing drug treatments. After treatment, the media was collected, centrifuged (1 min at 13,000 × g), and stored on ice. Samples were diluted 1:10 in reagent diluent and loaded onto plates precoated with capture antibody. The absorbance was measured at OD_450_ using a BioTek Cytation 3 plate reader, with wavelength correction applied at OD_540_.

### Wound scratch cell migration assays

Cells were seeded at a high confluency in 12-well plates (350,000 cells/well) and grown to full confluency overnight. Next, the cells were scratched in a straight vertical line using a 200 µL pipette tip to form a wound within the confluent cell layer. The media was subsequently aspirated before being replaced with serum starvation media (2% (v/v) FBS) containing the drug to be assayed or vehicle (DMSO). The wounds were immediately imaged via stereomicroscopy at 4x, and a pen marking was made for later reference of the area to be observed. At 24 and 48 h, the wounds were imaged again to visualise the extent to which the wounds were closed over time. The area of the wound scratch was calculated with ImageJ, and the extent of closure was recorded as a percentage.

### Quantitative reverse transcription PCR (qRT–PCR) analysis

*TSC2*(−) or *TSC2*(+) AML cells were grown to 70% confluency. Media was replaced with serum-depleted media for 24 h prior to cell collection in RNAprotect (Qiagen, West Sussex, UK) and then stored at − 80 °C. RNA was isolated using the RNeasy Plus Mini Kit (Qiagen, West Sussex, UK), and cDNA was generated with the Reverse Transcriptase Core Kit (Eurogentec, Belgium). qRT‒PCR was performed using TakyonTM ROX Sybr MasterMix dTTP blue (Eurogentec, Belgium). Ct values were normalised to IPO8 and TUBA1A. Primers were purchased from Integrated DNA Technologies and optimised for annealing temperature and efficiency. PDCD1LG2 forward primer GAACCCAGGACCCATCCAAC and reverse primer TTCAGATAGCACTGTTCACTTCCC and 183 bp amplicon length; IPO8 forward primer ACTGTTGCACATTGTTAGAG and reverse primer ACTTTGCCAAATATCTCAGC and 138 bp amplicon length; TUBA1A forward primer TCTTCCACCCTGAGCAACTT and reverse primer GGAAAACCAAGAAGCCCTGG and 159 bp amplicon length. Dissociation curves were generated to verify the specificity of the primer sets.

### Statistical analysis

Protein band intensities were quantified using ImageJ. (v1.53). Band intensity was normalised to that of β-actin. Fold changes were normalised to the DMSO control, where applicable. Normalised data were inputted into GraphPad Prism 9 (Dotmatics, Boston MA, USA), and statistical analysis was carried out. Normality testing in Prism 9 was carried out with D’Agostino and Pearson tests and the Shapiro‒Wilk test. Normally (Gaussian) distributed data were then analysed by ordinary one-way ANOVA with Tukey’s multiple comparisons or two-way ANOVA with Šídák’s multiple comparisons. When analysing 2 groups only, a parametric unpaired t test was carried out. All graphs represent data with ± Standard Error of the Mean (SEM). Non normally distributed data were assessed by the Kruskal‒Wallis test with Dunn’s multiple comparisons test. For comparisons between two groups, nonparametric Wilcoxon tests were used instead. *p* values: * < 0.05, ** < 0.01, *** < 0.001, **** (or #) < 0.0001, or not significant ‘NS’.

## Results

### *TSC2* loss is characterised by dysregulated expression of NF-κB genes

**Fig. 1:**
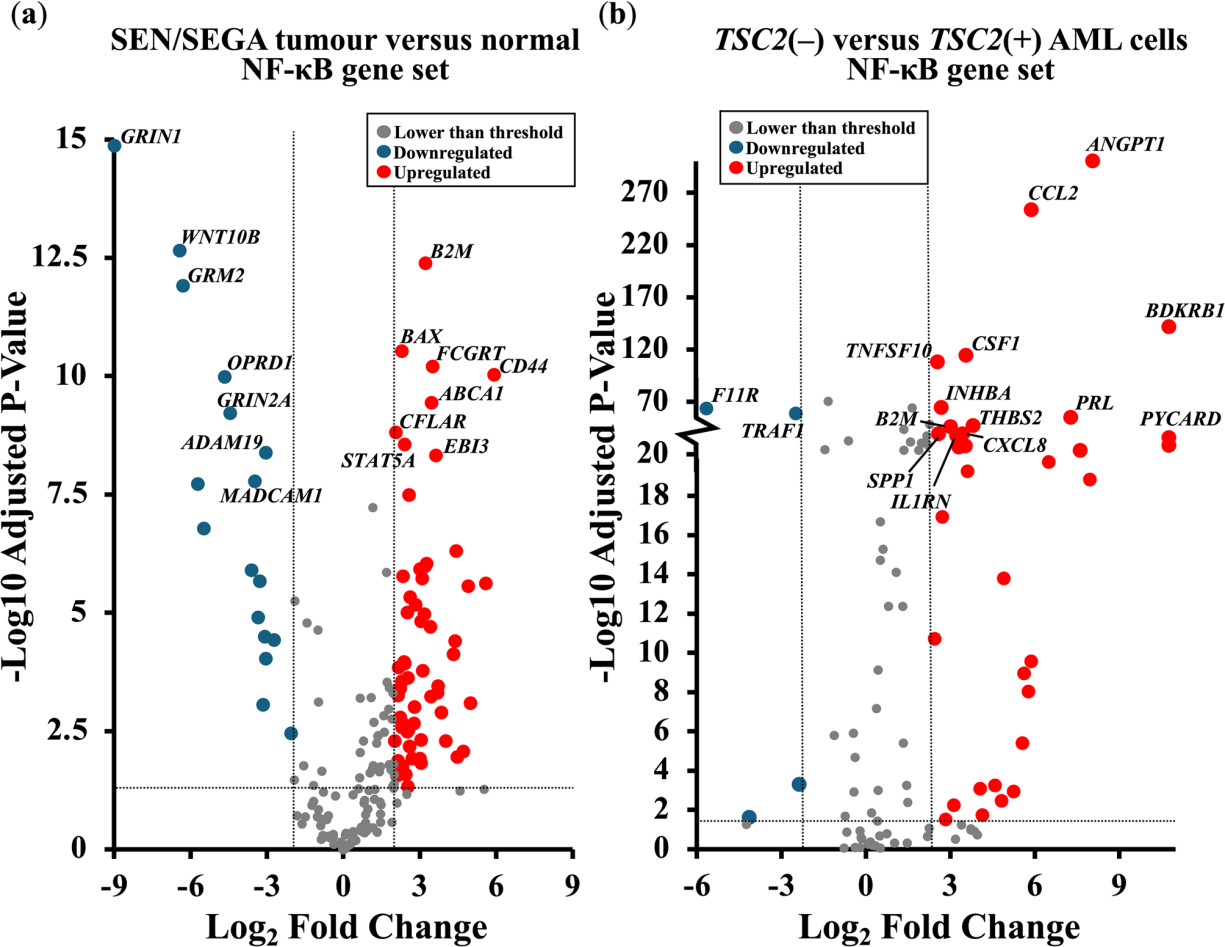
NF-κB regulatory and target genes are differentially expressed in TSC. Volcano plots showing the differential expression of NF-κB-linked genes in (**a**) TSC patient-derived brain tumours (*n* = 15) and (**b**) cultured *TSC2*(−) AML lacking functional TSC2 (*n* = 6), when compared to non-TSC brain tissue and *TSC2*-rescued *TSC2*(+) AML cells, respectively. The 15 most significantly dysregulated genes are labelled. Horizontal dashed lines represent an adjusted *p* value = 0.05; the vertical dashed lines represent a log_2_ fold change of ±2. Sample collection, analysis, and statistical analysis of the dataset used in the volcano plots were performed by Martin et al. [26].

To explore dysregulated gene expression in TSC, mRNA sequencing (RNAseq) data from 15 TSC patient SEN/SEGA samples were compared to non-TSC brain tissue (as previously described [26]), and RNAseq data from *TSC2*(−) AML cells (621 − 101) were compared to those from *TSC2*(+) AML cells (621 − 103). Gene Ontology analysis of the differentially expressed genes indicated enrichment of inflammatory and immune response genes within TSC patient-derived tumours (supplementary data), as previously described [26]. To better understand these dysregulated inflammatory pathways in TSC, we analysed the expression of 190 regulatory and NF-κB target genes. This NF-κB-linked gene set was adapted from a list developed by the Gilmore laboratory (Boston University) [27]. Volcano plots of the differentially expressed genes illustrate the dysregulation of NF-κB-linked genes in TSC patient-derived brain tumours (Fig. 1a) and in *TSC2*(−) AML cells (Fig. 1b) compared with their respective wild-type controls.

The observed transcriptional signature suggested a redox imbalance that could create a tumour microenvironment of oxidative stress and inflammation. Within both in vivo and in vitro datasets, NF-κB-related genes were significantly dysregulated. Within SEN/SEGAs, a total of 47 significantly upregulated NF-κB regulatory and target genes were observed (over a Log2_-_fold change of 2 and adjusted *p* value < 0.05) compared to 19 significantly downregulated genes (below a Log2-fold change of − 2 and adjusted *p* value < 0.05). This pattern of NF-κB dysregulation persisted within cortical tubers (17 NF-κB-linked genes increased and 2 decreased; supplementary data, Fig. 1) and *TSC2*(−) AML cells (34 NF-κB-linked genes increased and 4 decreased). As we observed a greater abundance of upregulated NF-κB-linked genes, we hypothesized that the NF-κB pathway was activated in TSC. To investigate this phenomenon, we next assessed the activity of the NF-κB pathway within in vitro TSC cell lines.

### Altered pathway regulation of NF-κB and STAT3 in TSC2-deficient cells

**Fig. 2:**
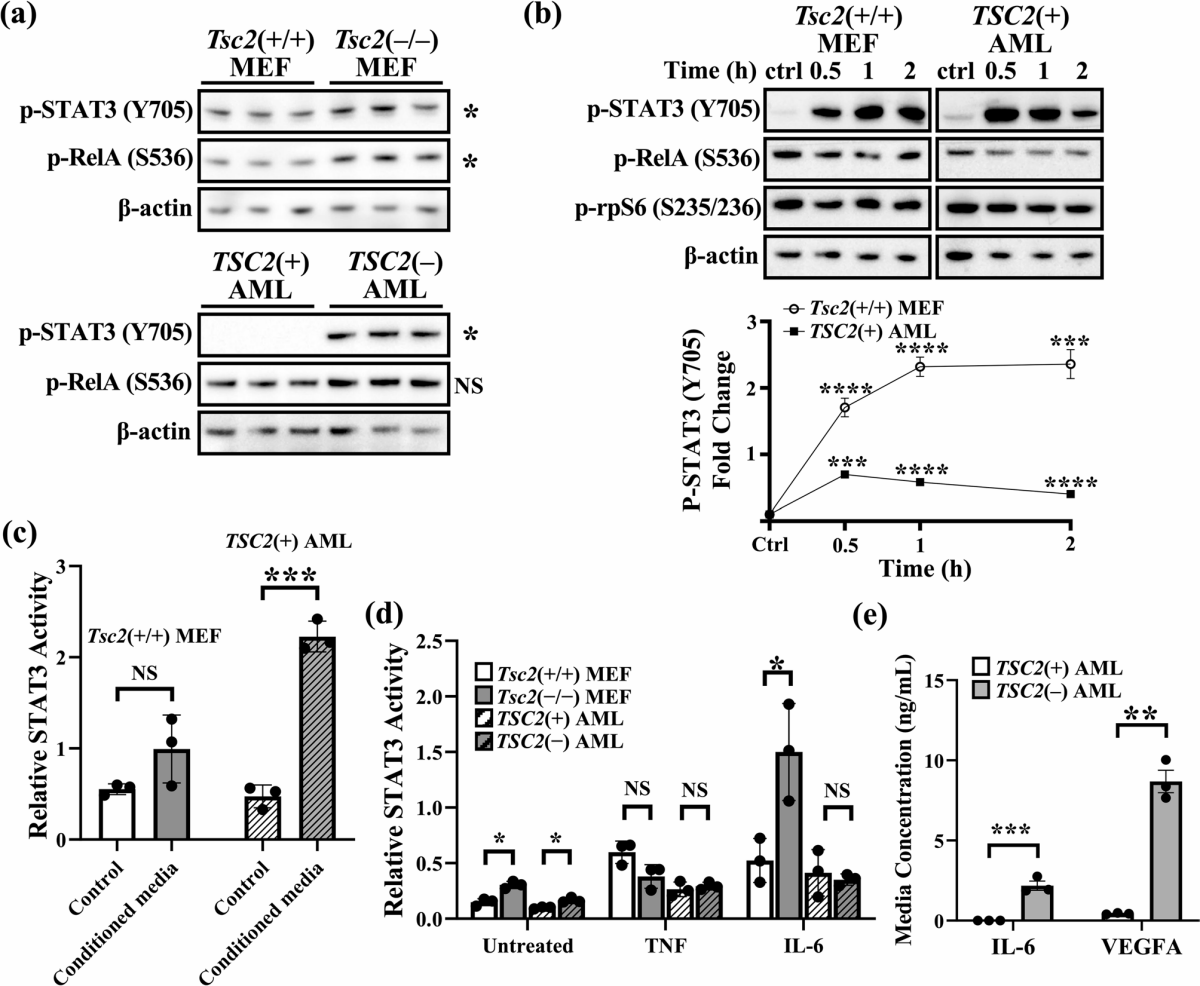
Complex NF-κB and STAT3 signalling interplay in TSC. (**a**) Confluent cells were serum-starved for 24 h and lysed. Western blot analysis of S536-phospho RelA and Y705-phospho STAT3 was carried out in *Tsc2*(−/−) MEFs (top panel) and *TSC2*(−) AML cells (bottom panel), respectively. β-actin was used as a loading control (the western blot panel shows *n *= 3; unpaired t test. Each lane represents one biological replicate, with all three biological replicates shown per condition.) (**b**) Serum-starved *Tsc2*(+/+) MEFs or *TSC2*(+) AML cells were stimulated with conditioned media from serum-starved* Tsc2*(−/−) MEFs or *TSC2*(−) AML cells, respectively, and western blot analysis of S536-phospho RelA, Y705-phospho STAT3, S235/236-phospho rpS6, and β-actin as a loading control was performed after 0.5, 1 and 2 h of treatment. Densitometric analysis of Y705-phospho-STAT3 is also shown (*n = *3 biological replicates; one-way ANOVA). (**c**) The control and 1 h treatment conditions from (**b**) were subjected to STAT3 transcription assays, and relative STAT3 nuclear activity is shown after treatment (*n *= 3, unpaired t test). (**d**) Serum-starved *Tsc2*(−/−) and *Tsc2*(+/+) MEFs and *TSC2*(−) and *TSC2*(+) AML cells were stimulated with 30 ng/mL TNF for 2 h or 50 ng/mL IL-6 for 1 h, as indicated. STAT3 activity assays were carried out to show relative STAT3 nuclear activity (*n *= 3; unpaired t tests). (**e**) The media concentration of IL-6 and VEGF-A were compared between *TSC2*(+) and *TSC2*(−) AML cells by ELISA(*n *=* 3*, unpaired t test). All graphs show error as ± SEM

STAT3 is a downstream target of NF-κB, and these two pathways are closely linked [11]. Previous research has indicated that STAT3 signalling is enhanced in *TSC2*-deficient cells [19, 28]. We sought to characterise the activity of NF-κB and STAT3, including cytokine responsiveness, in TSC cell models. For this purpose, we used *TSC2*(−) or *TSC2*(+) AML cells as well as *Tsc2*(+/+) or *Tsc2*(−/−) murine embryonic fibroblasts (MEFs). We observed increased phosphorylation of S536-RelA and Y705-STAT3 in both *Tsc2*(−/−) MEFs and *TSC2*(−) AML cells compared to their respective TSC2-expressing controls (Fig. 2a). As these phosphorylation sites are required for the activity of RelA and STAT3, these data imply that both NF-κB and STAT3 become more transcriptionally active upon the loss of *TSC2*. To explore potential autocrine signalling crosstalk to STAT3, wild-type control cells were stimulated with conditioned media from their respective untreated serum-starved *TSC2*-deficient cell lines (Fig. 2b), and STAT3/NF-κB pathway activation was assayed via western blotting. Supplementation with conditioned media (obtained from *TSC2*-deficient cells) caused acute STAT3 activation in both cell lines, suggesting that *TSC2*-deficient cells secrete factors that potently activate the STAT3 pathway. This was confirmed by STAT3 transcriptional activation ELISA, in which wild-type *Tsc2*(+/+) MEFs and *TSC2*(+) AML cells were treated with their corresponding *TSC2*-deficient cell conditioned media for 1 h, which strongly promoted STAT3 nuclear activation (Fig. 2c). Next, we tested whether TSC2 expression affects the cytokine-induced transcriptional activity of STAT3 using 2 h of TNF (30 ng/mL) or 1 h of IL-6 (50 ng/mL). While STAT3 activation after TNF and IL-6 stimulation was similar in *TSC2*(−) and *TSC2*(+) AML cells, *Tsc2*(−/−) MEFs were more sensitive to IL-6 treatment, in which STAT3 was induced 4.9-fold (Fig. 2 d). Conversely, *Tsc2*(+/+) MEFs exhibited a 3.5-fold increase in STAT3 activity following IL-6 stimulation. According to the STAT3 transcription assays, *Tsc2*(−/−) MEFs appeared to have a less significant response to TNF than did *Tsc2*(+/+) MEFs. Based on these data, we hypothesised that *TSC2*(−) AML cells release more cytokines, which in turn enhances inflammatory autocrine signalling. Using ELISA, we confirmed a > 19-fold increase in VEGF-A in conditioned media taken from *TSC2*(−) AML cells (Fig. 2e). IL-6 secretion was not detected in *TSC2*(+) AML cells but was significantly increased in *TSC2*(−) AML cells.

### NF-κB Inhibition reduces STAT3 activation

**Fig. 3:**
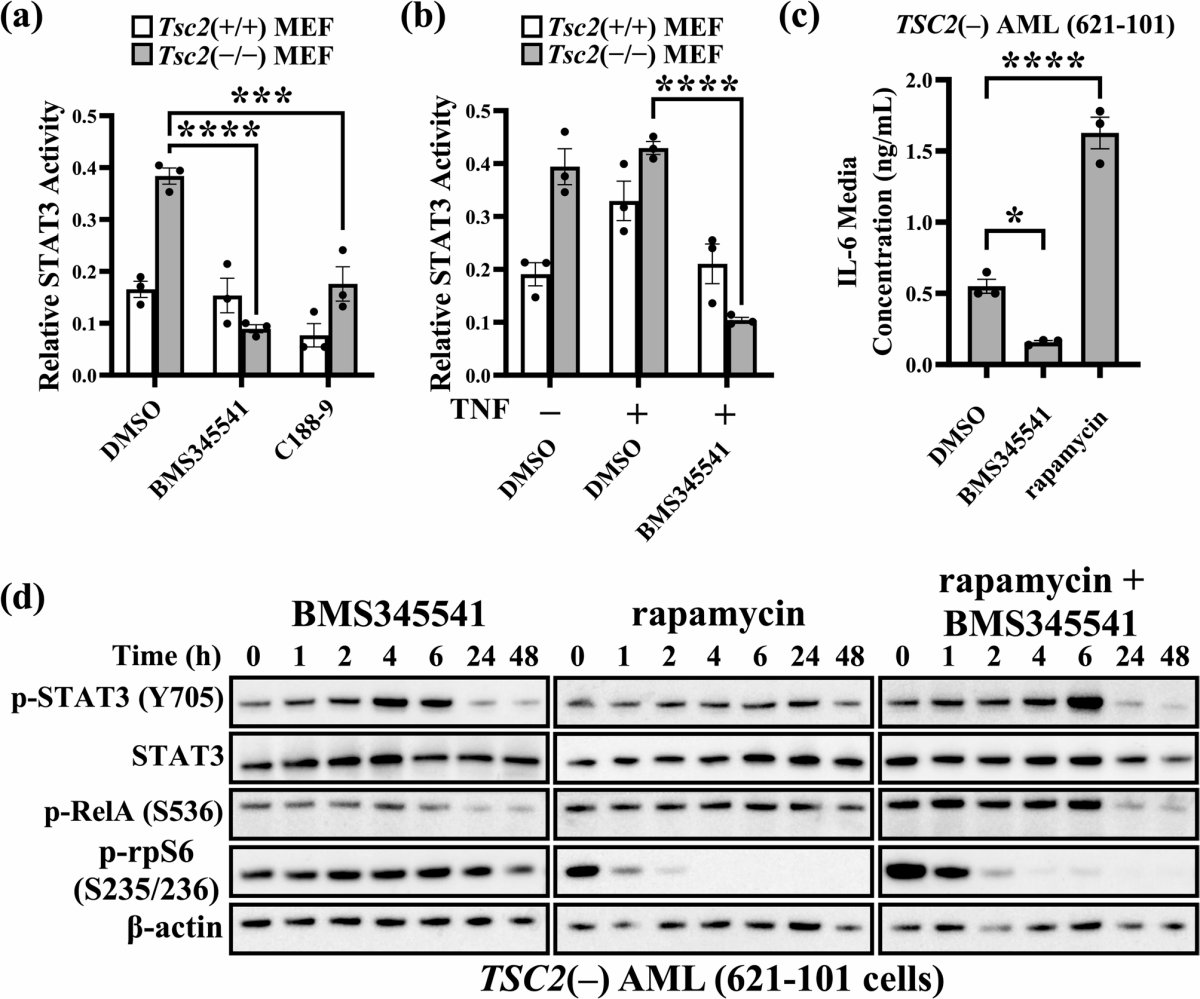
Autocrine signalling crosstalk between NF-κB and STAT3 in TSC cell models. Relative STAT3 activity was assessed in nuclear lysates prepared from *Tsc2*(−/−) and (+/+) MEFs treated with (**a**) DMSO (control), 5 μM BMS345541 or 15 μM C188-9 for 24 h or (**b**) TNF (30 ng/mL, 2 h treatment) after pretreatment with 5 μM BMS345541 for 24 h, as indicated (*n* =* 3*; two-way ANOVA with Tukey’s multiple comparisons). (**c**)*TSC2*(−) AML cells were treated with DMSO control, 10 μM BMS345541, or 50 nM rapamycin for 24 h. Conditioned media from these cells were subjected to IL-6 ELISAs (*n = 3;* one-way ANOVA with Tukey’s multiple comparisons). (**d**) The phosphorylation status of RelA, STAT3, and rpS6 was investigated over time (0, 1, 2, 4, 6, 24, and 48 h) with 5 μM BMS345541 or 50 nM rapamycin as single or combination treatments (*n* =* 3 *biological replicates). All graphs show error as ± SEM.

Next, we examined whether NF-κB inhibition could diminish the increase in STAT3 activity in *TSC2*-deficient cells. To do this, *Tsc2*(−/−) MEFs and *Tsc2*(+/+) MEFs were treated with 5 µM BMS345541 (an IKK complex allosteric inhibitor), after which the transcriptional activity of STAT3 was measured. In *Tsc2*(−/−) MEFs but not in Tsc2(+/+) MEFs, STAT3 activity was reduced after 24 h of BMS345541 treatment (Fig. 3a). As a control, we used C188-9, a STAT3 inhibitor, in both the *Tsc2*(−/−) and *Tsc2*(+/+) MEFs. C188-9 reduced STAT3 activity in both *Tsc2*(+/+) and *Tsc2*(−/−) MEFs. C188-9 and BMS345541 both reduced STAT3 activity to a similar extent, demonstrating that heightened NF-κB activity in *Tsc2*(−/−) MEFs may be responsible for the observed upregulation of STAT3. Furthermore, NF-κB inhibition by BMS345541 blocked the TNF-induced activation of STAT3 in *Tsc2*(+/+) MEFs (Fig. 3b), suggesting that NF-κB is required for cytokine-induced STAT3 induction. Since we previously demonstrated that *TSC2*-deficient cells secrete high levels of IL-6, it is possible that STAT3 activity and IL-6 secretion are linked. Furthermore, IL-6 was recently shown to be overexpressed in TSC2 disease models, and inhibition with IL-6 antagonists was shown to reduce tumour growth [29]. Therefore, we next aimed to investigate whether NF-κB inhibition could regulate IL-6 secretion. *TSC2*(−) AML cells were treated with 10 µM BMS345541 or 50 nM rapamycin for 24 h, after which IL-6 levels were measured via ELISA. BMS345541 reduced IL-6 secretion by approximately 4-fold, whereas rapamycin increased IL-6 secretion by approximately 3-fold, although the mechanism behind this is unknown (Fig. 3c). Next, we investigated how NF-κB inhibition may reduce STAT3 phosphorylation at Tyr705 after 48 h of treatment compared to that resulting from treatment with either rapamycin or a combination of both drugs. Rapamycin was used to determine the effects of mTORC1 inhibition on NF-κB and STAT3 activity. We also aimed to observe whether the impact of NF-κB inhibition on STAT3 activity was mTORC1 dependent. Surprisingly, we identified a biphasic response to NF-κB inhibition in *TSC2*(−) AML cells, with an initial increase in Y705-STAT3 phosphorylation that subsequently decreased at the 24- and 48-h time points (0.8-fold and 0.6-fold, respectively) (Fig. 3 d). Rapamycin had little effect on RelA or STAT3 phosphorylation but did ablate rpS6 phosphorylation, as expected. Moreover, combination treatment with BMS345541 and rapamycin dampened the increase in STAT3 phosphorylation at 6 h (3.2-fold increase for BMS345541 versus 1.58-fold increase for combination treatment). Combinatorial treatment with BMS345541 and rapamycin also reduced the total level of STAT3 at later timepoints, whereas BMS345541 treatment did not elicit this effect. At later time points (24 and 48 h), combination treatment with BMS345541 and rapamycin had the most potent reduction in STAT3 phosphorylation, although the mechanisms underpinning this remain unclear, and further work is required to understand the biphasic response (supplementary data, Fig. 2).

### NF-κB inhibition reduces anchorage-independent growth and cell migration in *TSC2*-deficient cells

**Fig. 4:**
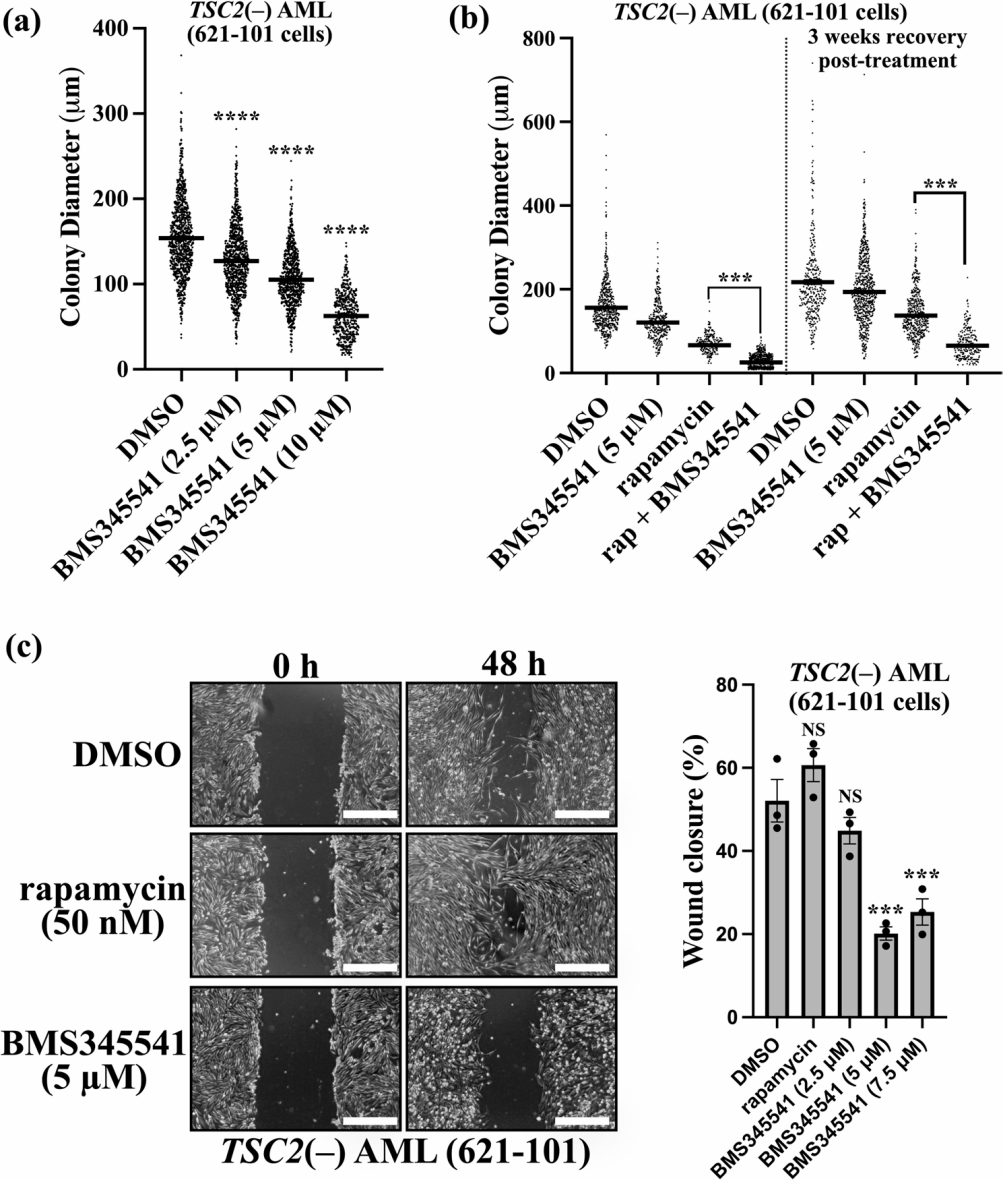
NF-κB inhibition reduces anchorage-independent growth and migration in *TSC2*-deficient cells. (**a**)*TSC2*(−) AML cells were grown for 3 weeks in soft agar supplemented with increasing concentrations of BMS345541 (0, 2.5, 5, or 10 μM), where indicated. Thirty phase contrast images were taken per condition, and the diameters of all the visible colonies were determined in ImageJ. (v1.53) [32]. (Kruskal‒Wallis test with Dunn’s multiple comparison tests.) (**b**) Same as for panel ‘**a**’ but with the inclusion of 50 nM rapamycin as a single or combination treatment with BMS345541. The colony diameter was recorded before the media was replaced with untreated media for an additional 3 weeks in the absence of drug. (**c**) Wound scratch assays were carried out on *TSC2*(−) AML cells in the presence of the DMSO vehicle only control, rapamycin (50 nM) or BMS345541 (5 μM). The ‘wound’ was imaged after 48 h, and wound closure (%) was calculated using ImageJ. (v1.53). Representative images are shown, 500 μm scale bar (two-way ANOVA with Tukey’s multiple comparison tests, *n* = 3). Error is shown as ± SEM.

To explore whether NF-κB inhibition might limit tumourigenesis, in vitro colony growth assays were carried out. Colonies of *TSC2*(−) AML cells were grown over 3 weeks in soft agar supplemented with increasing doses of BMS345541. At 10 µM, BMS345541 reduced anchorage-independent growth nearly 3-fold (Fig. 4a). Additionally, compared with DMSO treatment, 10 µM BMS345541 treatment reduced the number of colonies by half (989 versus 538 colonies). As TSC patient tumours regrow after the discontinuation of mTORC1 inhibitors [10], rapamycin was also compared as a single-drug treatment and in combination with BMS345541 (Fig. 4b). Anchorage-independent growth was assessed after 3 weeks of drug treatment. Overall, reduced colony growth was observed in the presence of BMS345541, and the combination of BMS345541 and rapamycin had a more potent effect on colony growth. To explore drug recovery, anchorage-independent growth was further evaluated after removal of the drug for an additional 3 weeks. Importantly, combined treatment with BMS345541 and rapamycin markedly reduced anchorage-independent growth upon discontinuation of treatment, which was more effective than treatment with rapamycin alone. Anchorage-independent growth assays were also performed with both the MEF and ELT3 TSC cell lines, which exhibited similar trends of colony growth reduction in response to the NF-κB inhibitor (supplementary data, Fig. 3).

Finally, we investigated the effects of NF-κB inhibition on the migration of *TSC2*(−) AML cells. NF-κB is known to influence migration and metastasis in cancers [30], and migration is also a key feature of lymphangioleiomyomatosis (LAM), which can occur in patients with TSC [31]. To do this, wound scratch assays were carried out in reduced-serum media supplemented with BMS345541 for two days. We observed a reduction in migration in cells treated with BMS345541, whereas rapamycin was ineffective at reducing migration (Fig. 4c).

### The immune checkpoint protein PD-L2 is dysregulated in TSC via inflammatory signalling

**Fig. 5:**
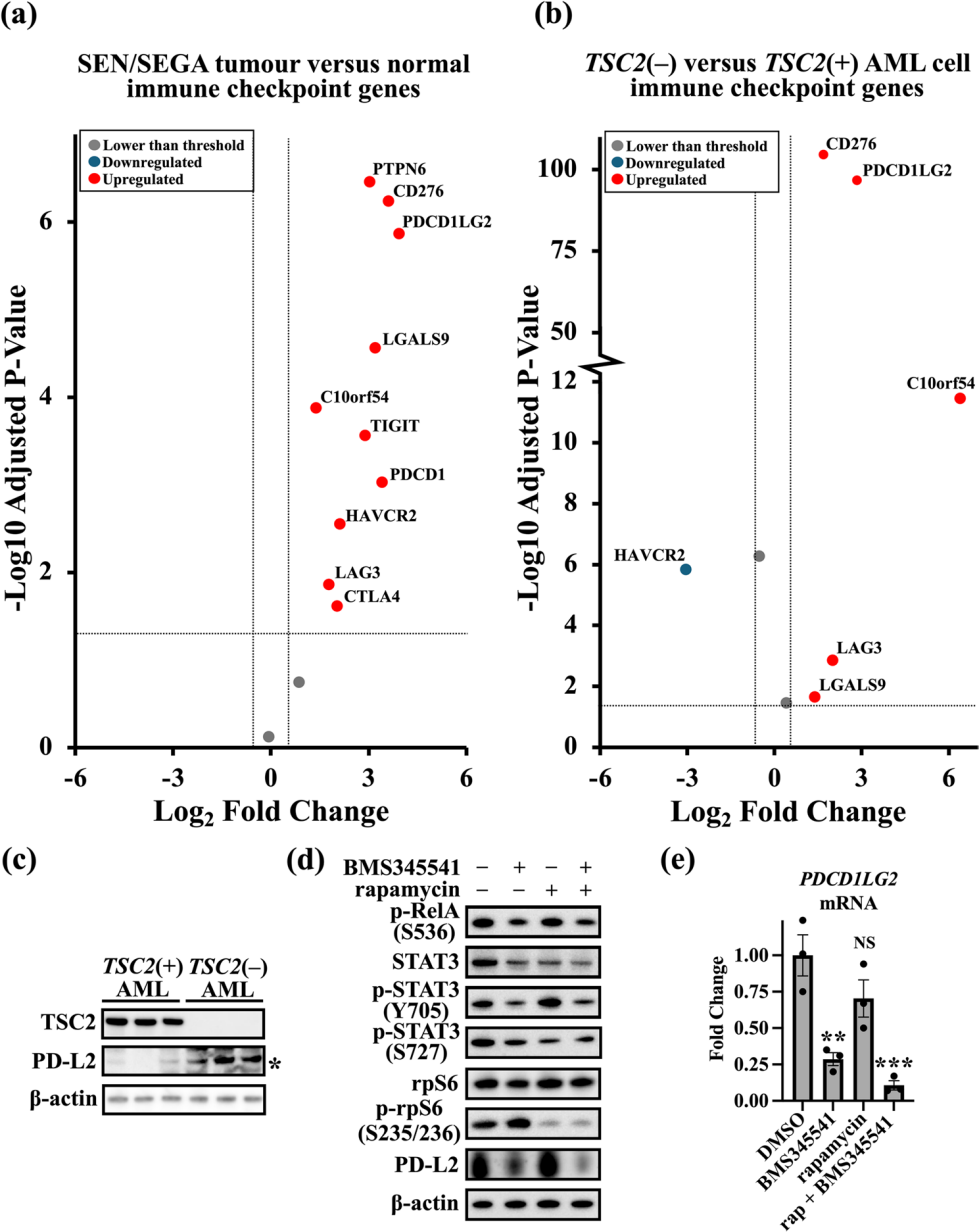
Immune checkpoint genes are dysregulated in TSC. Volcano plots showing the differential expression of immune checkpoint genes in (**a**) TSC patient-derived brain tumours (*n* = 15) and (**b**) cultured *TSC2*(−) AML tissues lacking functional TSC2 (*n* = 6) compared to non-TSC brain tissue and *TSC2*-rescued *TSC2*(+) AML cells. All significantly dysregulated genes are labelled. Horizontal dashed lines represent an adjusted *p* value = 0.05; the vertical dashed lines represent a log_2_ fold change of ±2. Sample collection, analysis, and statistical analysis of the dataset used in the volcano plots were performed by Martin et al. [26]. (**c**) PD-L2 expression was compared between *TSC2*(−) and *TSC2*(+) AML cells (the western blot panel shows *n*= 3; unpaired t test. Each lane represents one biological replicate. Related experiments were also carried out which showed similar trends) (**d**) Serum-starved *TSC2*(−) AML cells were treated with vehicle only (DMSO), BMS345541 (5 µM), rapamycin (50 nM), or a combination of both drugs for 24 h, after which protein phosphorylation and expression were assessed. Densitometric analysis was performed after normalisation to the level of β-actin (*n *= 3; one-way ANOVA with Tukey’s multiple comparison tests). (**e**) Serum-starved *TSC2*(−) AML cells were treated with vehicle only (DMSO), BMS345541 (5 mM), rapamycin (50 nM), or a combination of both drugs for 24 h, and *PDCD1LG2 *expression was analysed via RT‒qPCR. The expression levels were normalised to those of *IPO8 *and *TUBA1A* (*n *= 3; one-way ANOVA with Tukey’s multiple comparisons test). Error is shown as ± SEM.

Inflammatory signalling via NF-κB can influence leukocyte recruitment and modulation, which is a disease facet that has been reported in TSC patient tumours [26]. Given these connections between TSC and immune signalling, we next compared the differential expression of immune checkpoint genes in both SEN/SEGA (Fig. 5a) and *TSC2*(−) AML cells (Fig. 5b). This set of immune checkpoint regulators was adapted from a list on ACROBiosystems [33]. Notably, we observed increased expression of *PDCD1LG2*, which is a negative regulator of T cells that can be expressed on stromal and/or tumour cells to repress immune recognition [34]. PD-L2 protein expression was markedly enhanced in *TSC2*(−) AML cells compared to that in the wild-type control cells (Fig. 5c), and PD-L2 expression was ablated when NF-κB was inhibited with 5 µM BMS345541 (Fig. 5 d). Inhibition of mTORC1 with rapamycin did not reduce the increase in the protein expression of PD-L2 in these TSC-affected cells. Similarly, the gene expression of *PDCD1LG2* was reduced after treatment with 5 µM BMS345541 but not after inhibition of mTORC1 with rapamycin (Fig. 5e). This study focused on PD-L2, however the investigation could be diversified to include other immune checkpoint regulators in further work.

## Discussion

The NF-κB pathway plays a key role in the progression of many cancers and inflammatory conditions via the upregulation of proinflammatory genes. Although inflammation is a known feature of TSC-associated tumours, the role that NF-κB plays in the disease pathology of TSC is poorly understood. This study aimed to elucidate the role of NF-κB in TSC and thus identify the potential role of NF-κB signalling in TSC pathogenesis. Our findings showed that NF-κB is dysregulated in TSC patient tumours and cell line models. Our data imply that mTORC1 inhibitor therapies are unlikely to ameliorate inflammation in TSC patients, suggesting that NF-κB dysregulation could contribute to the failure of current mTORC1 inhibitors to completely ameliorate TSC symptoms [10].

This study highlights that dysregulated NF-κB and STAT3 signalling contributes to the observed inflammatory signature found in TSC cell line models and in TSC patient tumours. These inflammatory signals may be linked to TSC-associated symptoms. For instance, neuroinflammation is linked to a variety of neuropsychiatric conditions, including TSC-associated neuropsychiatric disorders (TANDs) and neurodegenerative disorders. Neuroinflammation has also been characterised in schizophrenia and depression [35, 36]. A review by Matta et al. highlighted the prevalence of neuroinflammation in individuals with autism spectrum disorders [37], while a review by Aronica and Crino categorises the dominant role of neuroinflammation in epilepsy [38]. As hyperactivation of STAT3 is a known driver of epilepsy [39, 40], STAT3 (and NF-κB) might be connected to the neurological symptoms associated with TSC. Cortical tubers are a suspected focal point of epilepsy in patients with TSC. Inflammation through NF-κB activity may contribute to epileptogenic signalling. This is supported by enhanced NF-κB dysregulation in cortical tubers (supplementary data, Fig. 1).

NF-κB and STAT3 are closely linked to multiple mechanisms of signalling crosstalk (reviewed in [11]). The complex signalling interplay between NF-κB and STAT3 is evident and may partially explain the observed variation in the state of NF-κB activity in related TSC research studies [18]. Many cytokines are NF-κB responsive and these include IL-6 [41]. Consequently, NF-κB may indirectly activate STAT3 via a positive feedback loop, where IL-6 secretion will induce STAT3 activation via interleukin receptors. Signalling crosstalk between NF-κB and STAT3, which is a feature shared in cancer [42], including glioma [43], was apparent in cell line models of TSC. Inhibition of one component in this feedback loop may assist in dampening this inflammatory signal. Arguably, mTORC1 activation has been shown to contribute to NF-κB signalling, so standard therapy with mTORC1 inhibitors in TSC patients should have some capacity to prevent the inappropriate activity of NF-κB [44]. With our cell line models, we showed that secreted cytokines such as IL-6 likely contribute to STAT3 activation in TSC. NF-κB inhibition could reduce STAT3 activity in *TSC2*-deficient cells likely through the inhibition of IL-6 signalling. Rapamycin was ineffective at reducing IL-6 secretion and STAT3 activity in *TSC2*-deficient cell lines. Combinatorial treatment comprising mTORC1 inhibition and NF-κB inhibition was sufficient to reduce STAT3/NF-κB and mTORC1 signalling. However, it is important to note that in the cell line studies presented here, treatment with mTORC1 inhibitors was only carried out over short time periods (up to 3 days). It is possible that a longer duration of mTORC1 inhibition is required to reduce chronic inflammation in TSC-associated tumours and/or neuroinflammation, and this was not captured within the in vitro experimentation of this study. Supporting this line of thought, everolimus (a rapalogue) shows greater efficacy in TSC patients in reducing seizures after longer durations of treatment, i.e., up to 3 years of treatment [9].

Rapamycin is a cytostatic drug that can stabilise TSC-related disease. Through anchorage-independent growth assays, we demonstrated the cytostatic property of rapamycin. While rapamycin causes a marked reduction in growth, cell colonies quickly recover and grow after the end of rapamycin treatment. While single-drug inhibition of NF-κB had little long-term effectiveness in repressing the anchorage-independent growth of *TSC2*(−) AML cells, we observed a marked reduction in colony size with combined treatment with NF-κB/mTOR inhibitors, which persisted after removal of both drugs.

Finally, we aimed to identify dysregulated targets that were insensitive to mTORC1 inhibition. A high degree of immune cell infiltration likely contributes to the disease pathology of TSC; however, TSC-derived tumours appear to avoid being attacked by the immune system. This is likely due to upregulated immune checkpoint regulators, such as PD-L2, that can be expressed on stromal and/or tumour cells. Other studies have shown that STAT3 signalling can upregulate PD-L2 [45, 46]. In this study, we showed that STAT3 activity was linked to NF-κB activity in *TSC2*-deficient cells. Our findings indicate that PD-L2 expression could be downregulated by NF-κB inhibition in *TSC2*-deficient cells, which could be due to the inhibition of STAT3. Our data showed that combinatorial inhibition of NF-κB and mTORC1 is effective at inhibiting both mTORC1-sensitive and mTORC1-insensitive targets. However, further investigations are needed to determine whether other immune checkpoint regulators may also be regulated through dysregulated inflammatory signalling in TSC. This work suggested that combination therapy targeting both NF-κB and mTORC1 might have longer lasting benefits for treating tumours in patients with TSC.

## Conclusions

NF-κB signalling is dysregulated and likely contributes to inflammation/immune signalling in TSC. Dysregulated inflammatory/immune signalling cascades are not directly regulated by mTORC1 but may be restored via NF-κB pathway inhibitors. Therefore, the NF-κB signalling pathway may be a therapeutic target for the treatment of TSC, and combination approaches with traditional mTORC1 inhibitors may prove more effective as an adjunctive therapy. Pre-clinical and clinical trials are required to assess the efficacy of NF-κB pathway inhibitors in the treatment of TSC.

## Supplementary Information

Supplementary Material 1.

Supplementary Material 2.

Supplementary Material 3.

Supplementary Material 4.

Supplementary Material 5.

Supplementary Material 6.

## Data Availability

Raw data for RNAsequencing of TSC patient tumours was previously deposited in the Database of Genotypes and Phenotypes (dbGaP) under the accession code phs001357.v1.p1 [26]. All datasets generated or analysed during this study are either included in this article or supplementary files. The data analysed during the current study are available from the corresponding author upon reasonable request.
