# Supplementary material for: Loss of tuberous sclerosis complex 2 confers inflammation via dysregulation of nuclear factor kappa-light-chain-enhancer of activated B cells
Source: J Inflamm (Lond). 2025 Sep 26;22:38. doi: 10.1186/s12950-025-00464-8 (PMC12465316; doi:10.1186/s12950-025-00464-8)
Supplement: Supplementary file 4 — Supplementary Material 4. [file 12950_2025_464_MOESM4_ESM.pptx]

## Slide 1
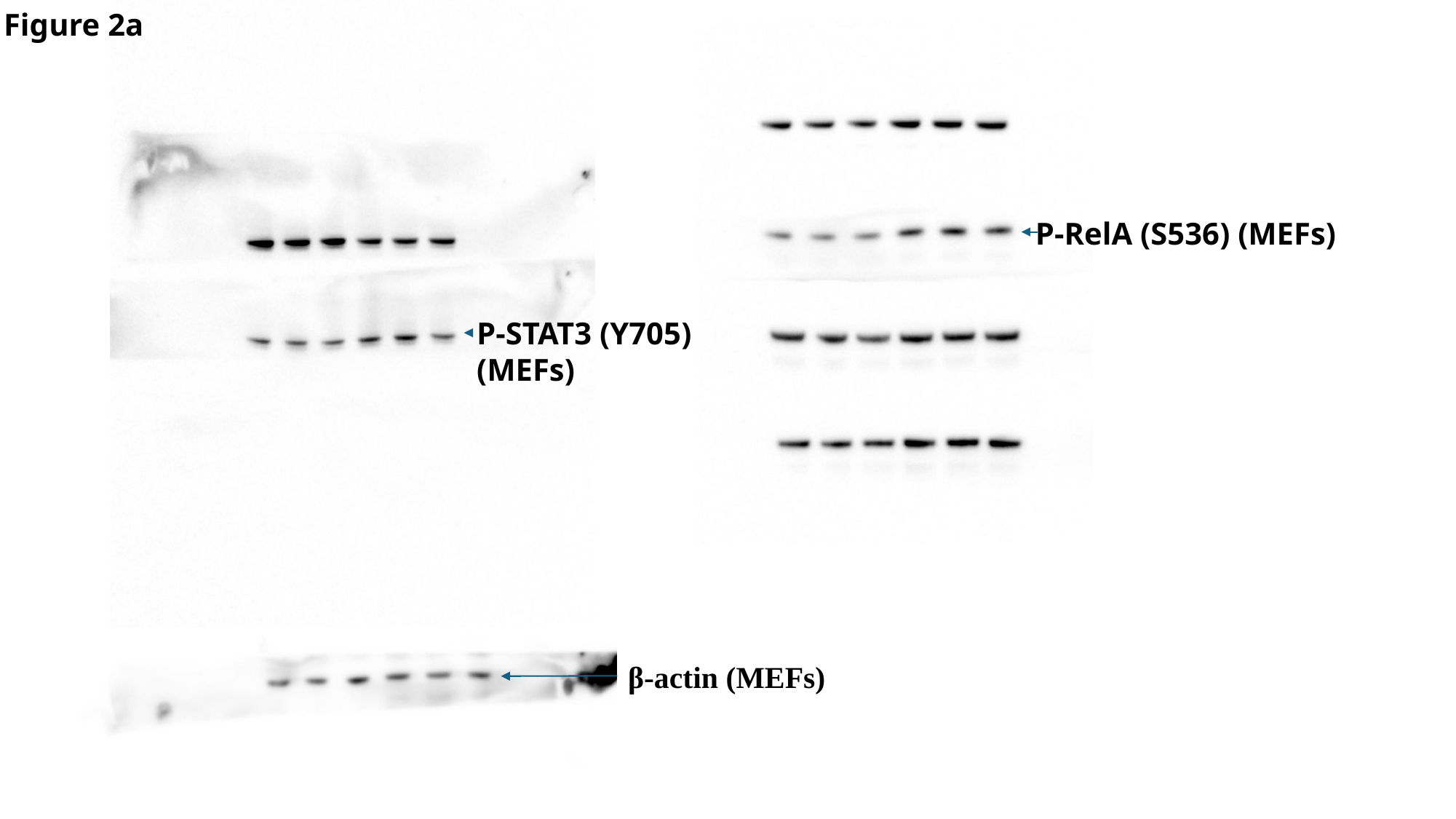

Figure 2a
P-RelA (S536) (MEFs)
P-STAT3 (Y705)
(MEFs)
β-actin (MEFs)

## Slide 2
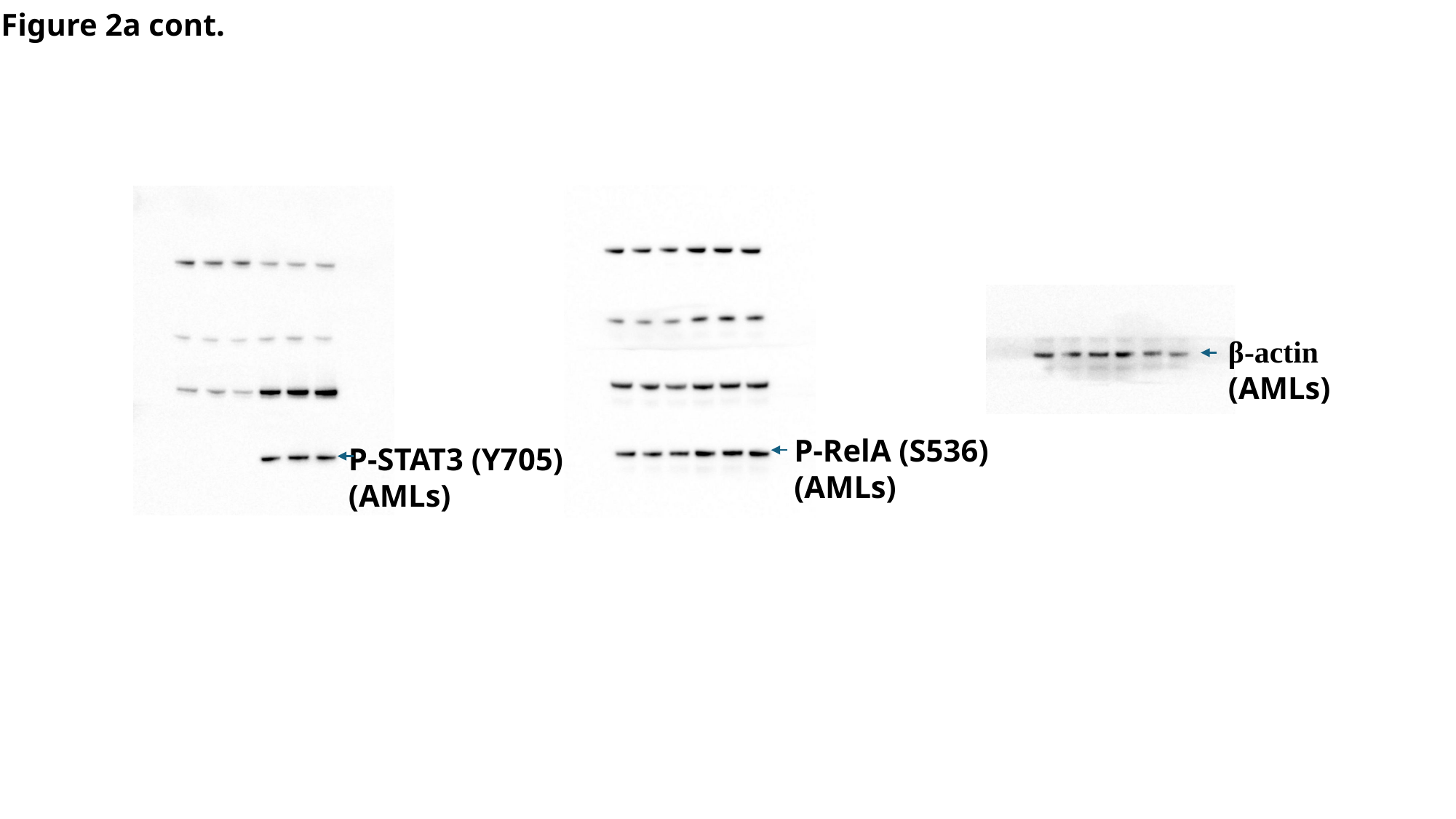

Figure 2a cont.
β-actin
(AMLs)
P-RelA (S536)
(AMLs)
P-STAT3 (Y705)
(AMLs)

## Slide 3
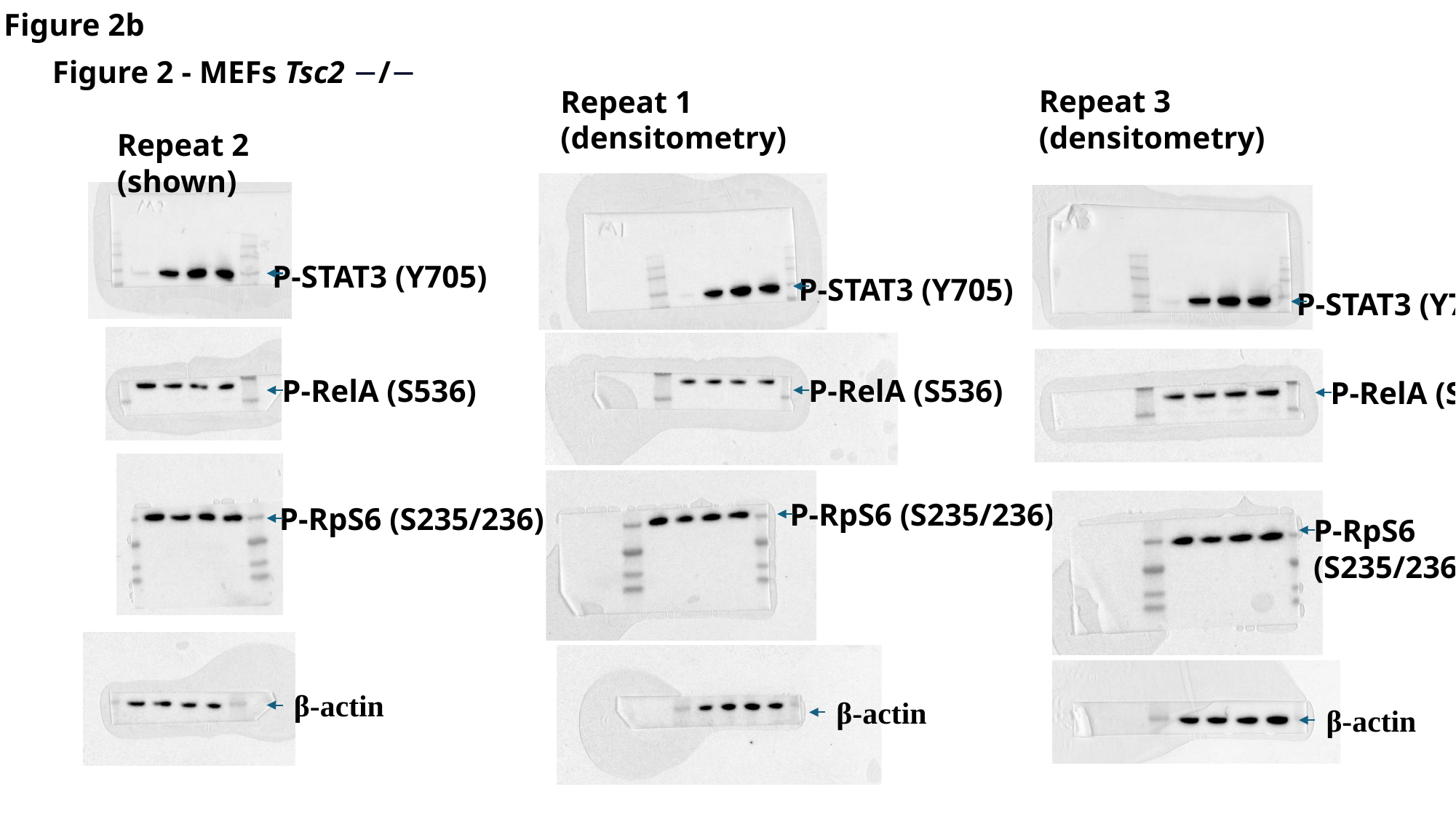

Figure 2b
Figure 2 - MEFs Tsc2 −/−
Repeat 3 (densitometry)
Repeat 1 (densitometry)
Repeat 2 (shown)
P-STAT3 (Y705)
P-STAT3 (Y705)
P-STAT3 (Y705)
P-RelA (S536)
P-RelA (S536)
P-RelA (S536)
P-RpS6 (S235/236)
P-RpS6 (S235/236)
P-RpS6
(S235/236)
β-actin
β-actin
β-actin

## Slide 4
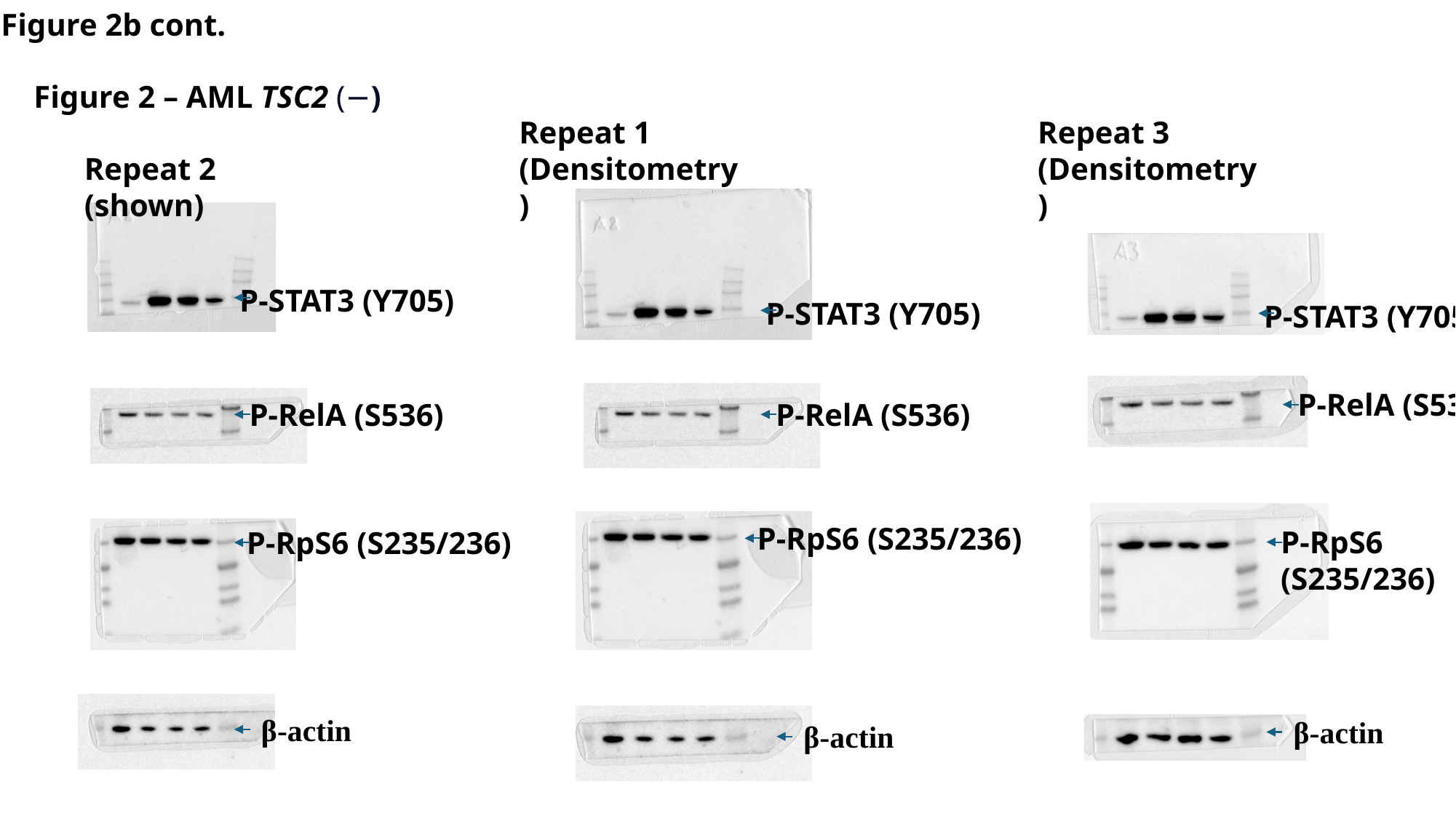

Figure 2b cont.
Figure 2 – AML TSC2 (−)
Repeat 1 (Densitometry)
Repeat 3 (Densitometry)
Repeat 2 (shown)
P-STAT3 (Y705)
P-STAT3 (Y705)
P-STAT3 (Y705)
P-RelA (S536)
P-RelA (S536)
P-RelA (S536)
P-RpS6 (S235/236)
P-RpS6
(S235/236)
P-RpS6 (S235/236)
β-actin
β-actin
β-actin

## Slide 5
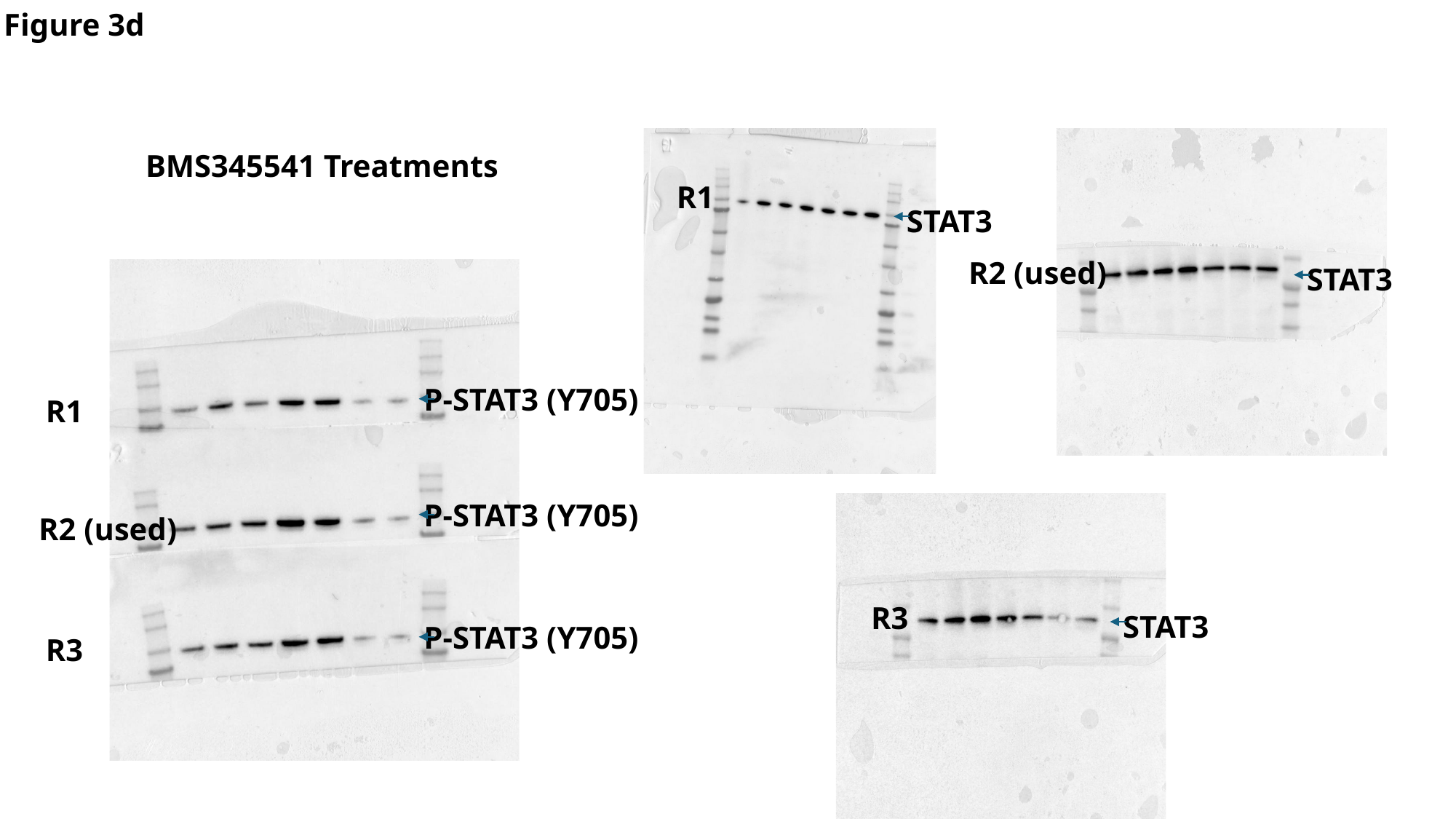

Figure 3d
BMS345541 Treatments
R1
STAT3
R2 (used)
STAT3
P-STAT3 (Y705)
R1
P-STAT3 (Y705)
R2 (used)
R3
STAT3
P-STAT3 (Y705)
R3

## Slide 6
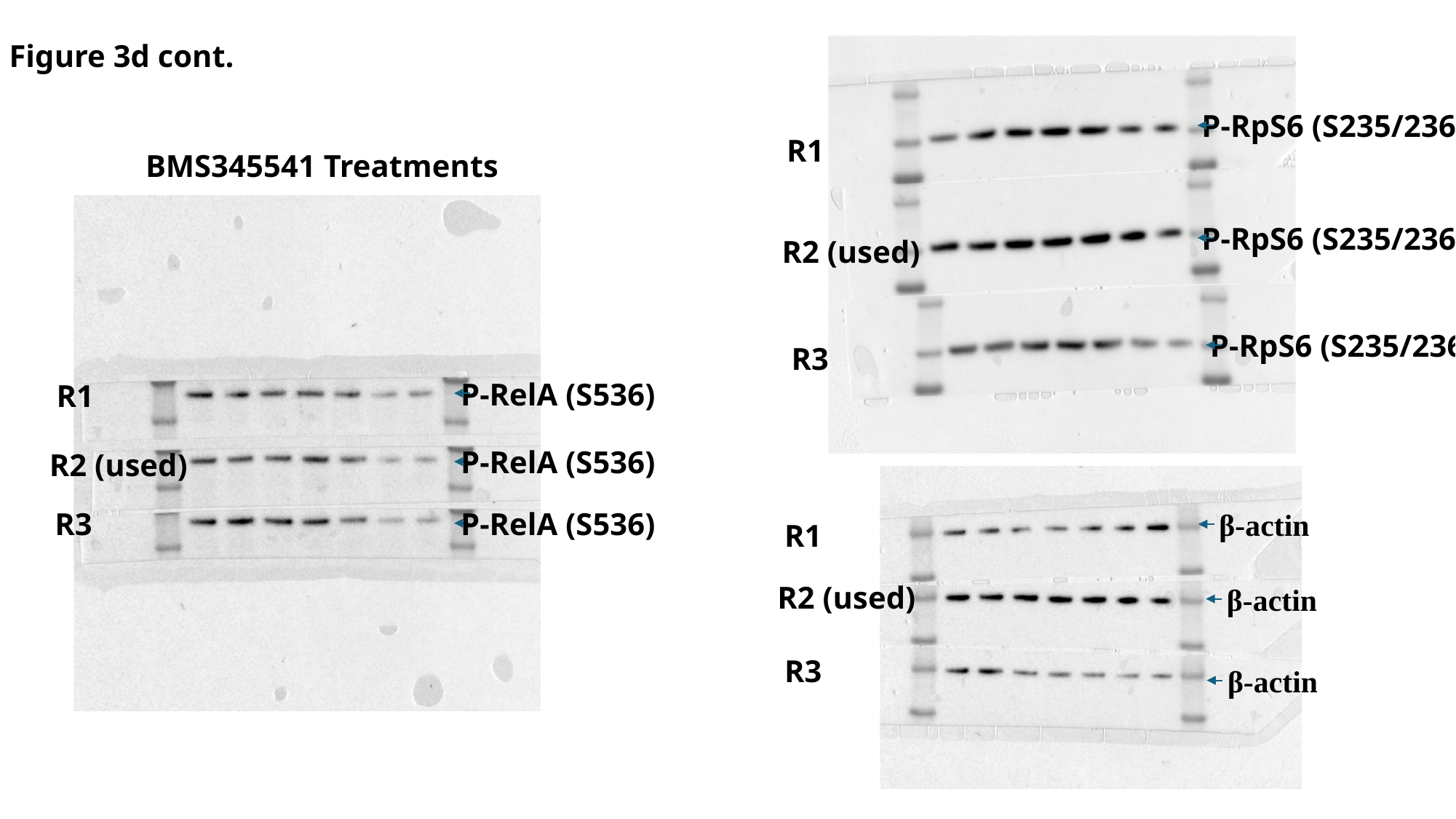

Figure 3d cont.
P-RpS6 (S235/236)
R1
BMS345541 Treatments
P-RpS6 (S235/236)
R2 (used)
P-RpS6 (S235/236)
R3
P-RelA (S536)
R1
P-RelA (S536)
R2 (used)
R3
P-RelA (S536)
β-actin
R1
R2 (used)
β-actin
R3
β-actin

## Slide 7
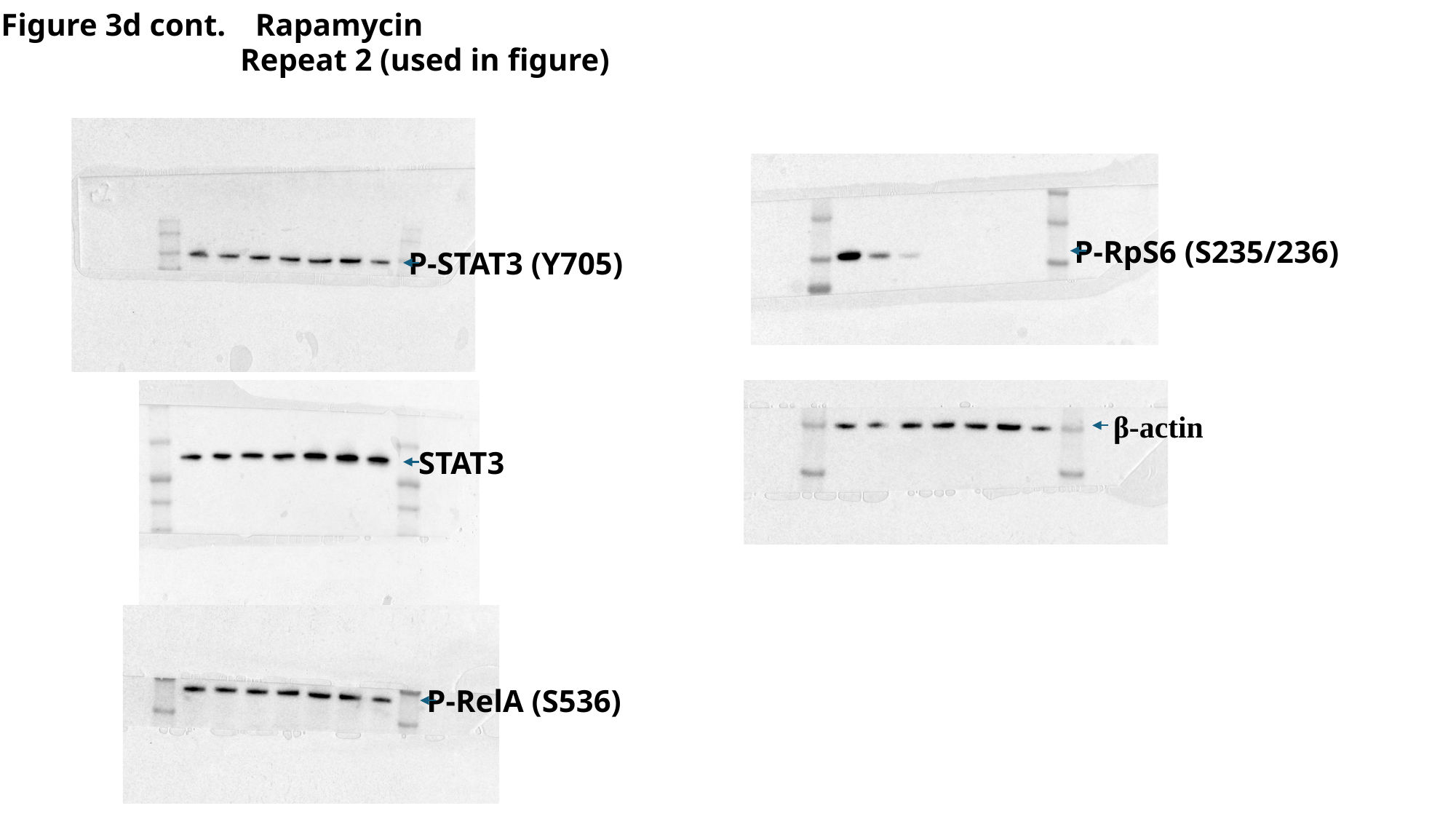

Figure 3d cont.
Rapamycin
Repeat 2 (used in figure)
P-RpS6 (S235/236)
P-STAT3 (Y705)
β-actin
STAT3
P-RelA (S536)

## Slide 8
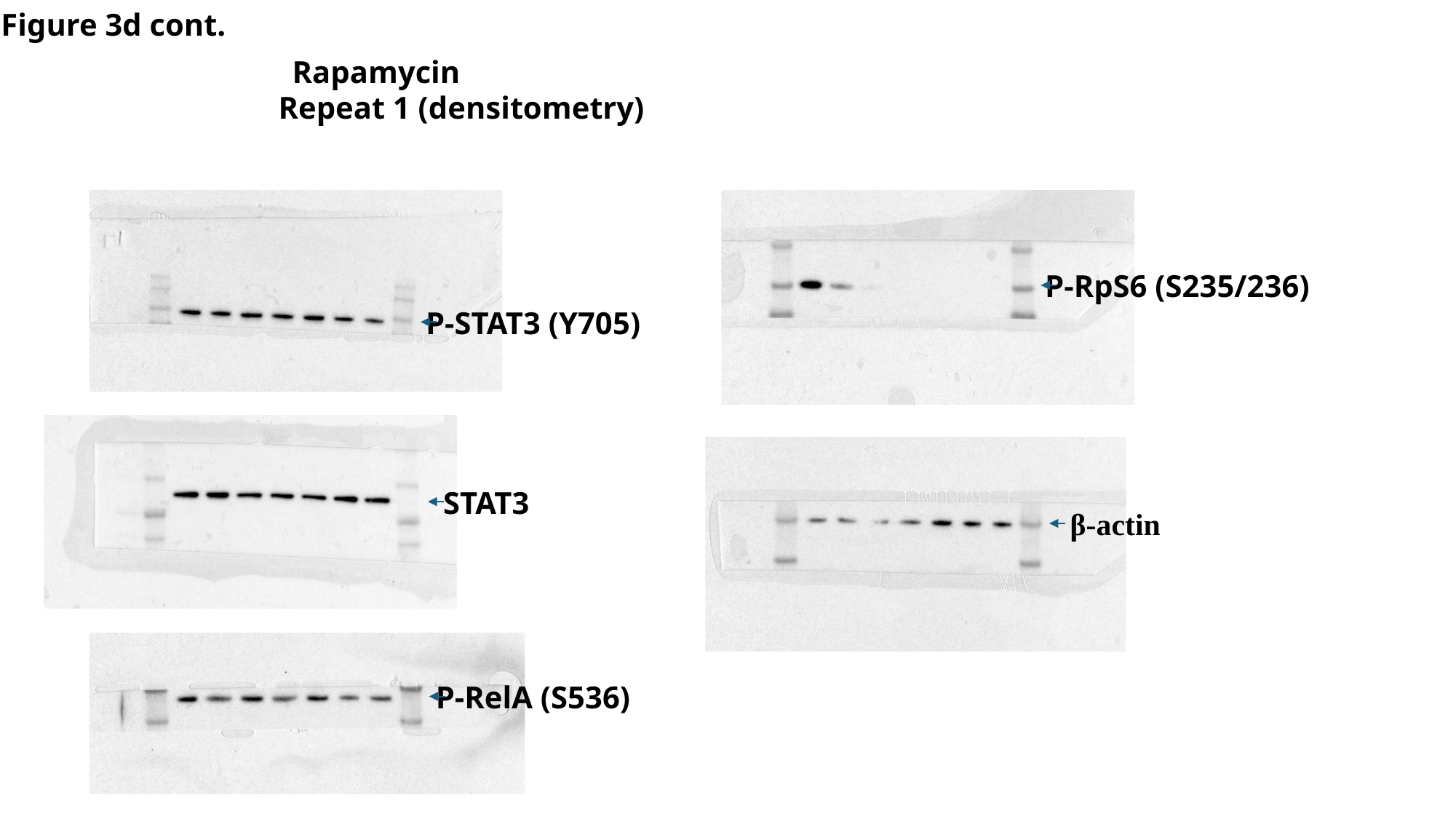

Figure 3d cont.
Rapamycin
Repeat 1 (densitometry)
P-RpS6 (S235/236)
P-STAT3 (Y705)
STAT3
β-actin
P-RelA (S536)

## Slide 9
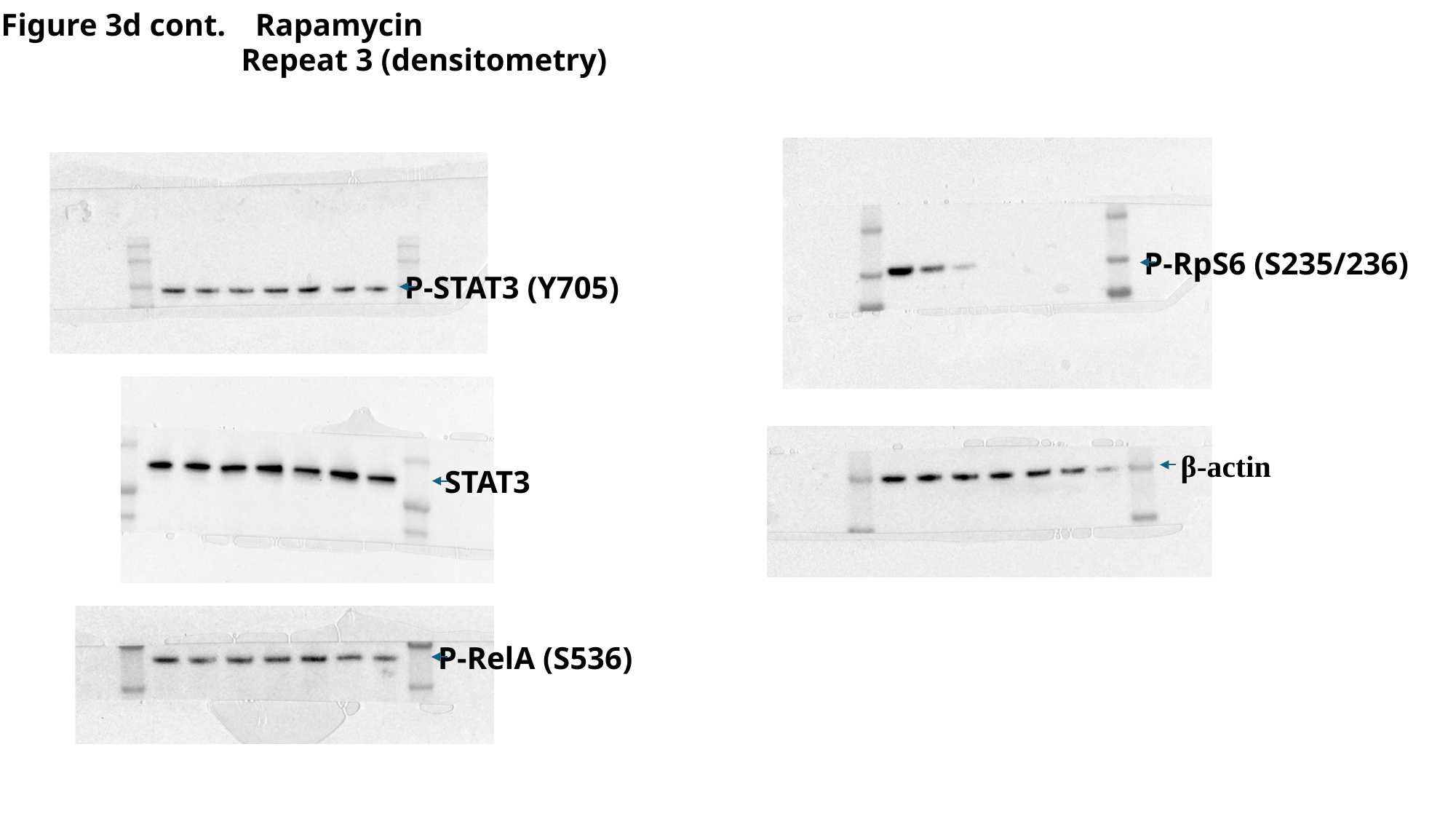

Figure 3d cont.
Rapamycin
Repeat 3 (densitometry)
P-RpS6 (S235/236)
P-STAT3 (Y705)
β-actin
STAT3
P-RelA (S536)

## Slide 10
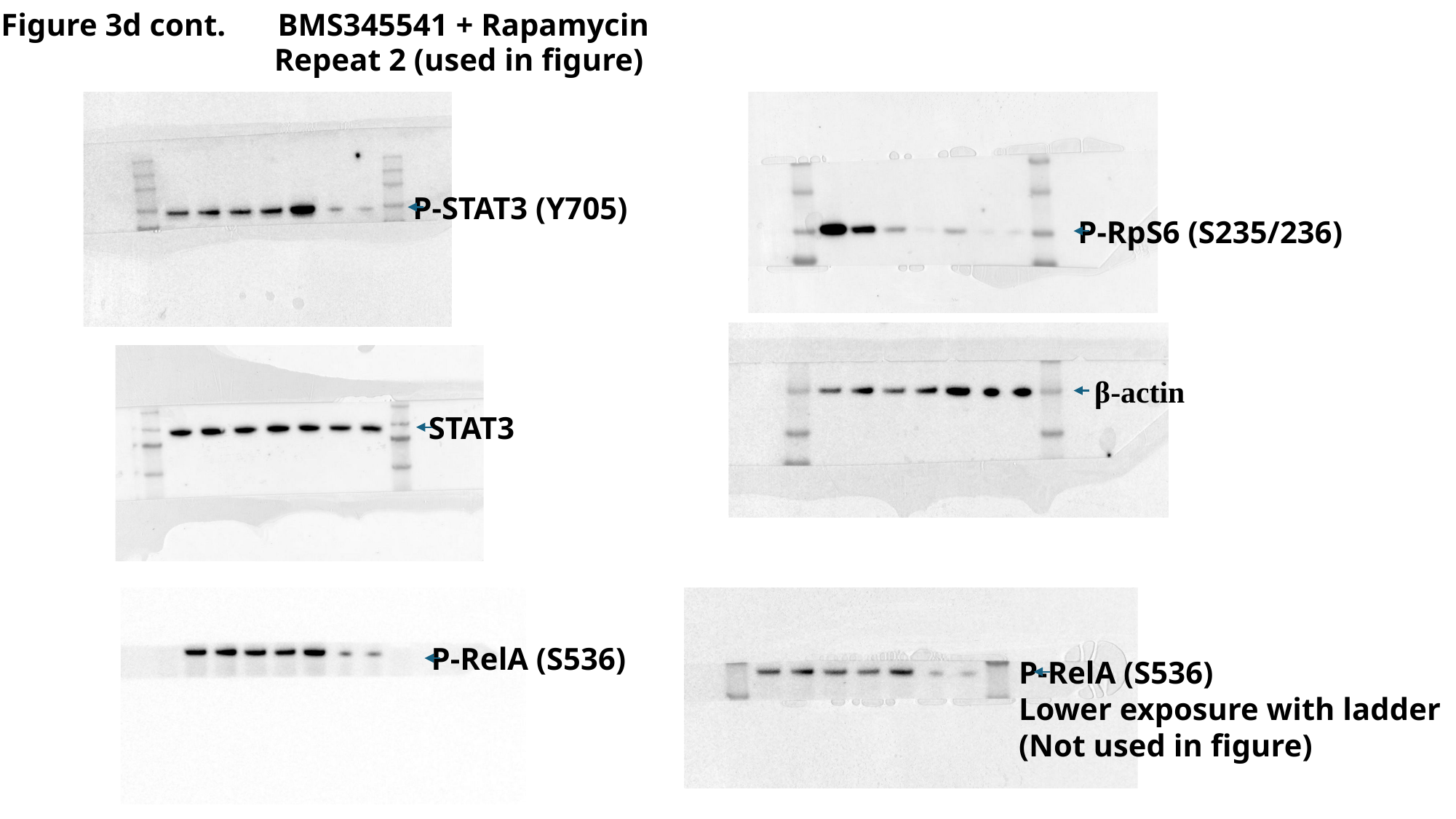

Figure 3d cont.
BMS345541 + Rapamycin
Repeat 2 (used in figure)
P-STAT3 (Y705)
P-RpS6 (S235/236)
β-actin
STAT3
P-RelA (S536)
P-RelA (S536)
Lower exposure with ladder
(Not used in figure)

## Slide 11
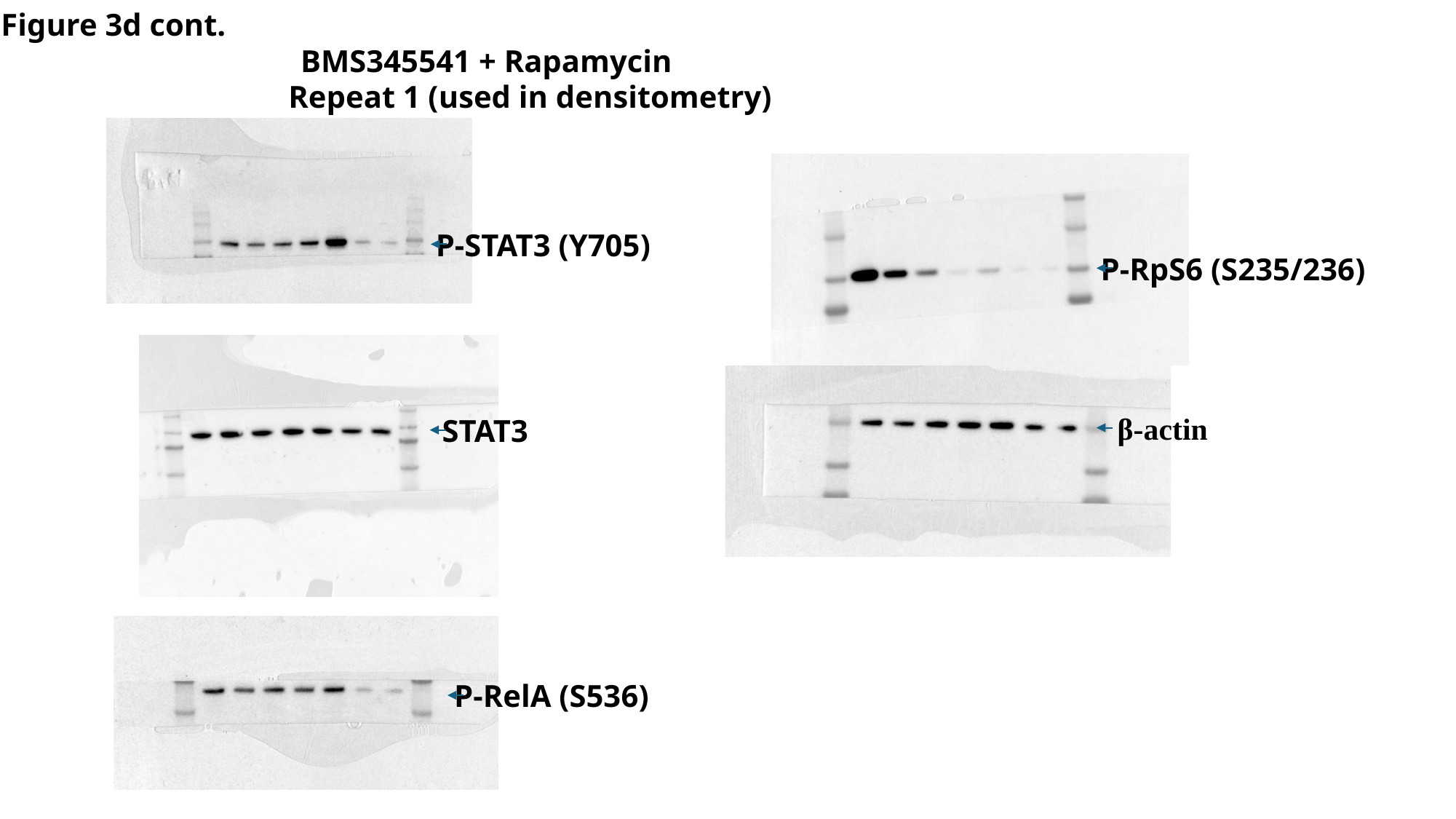

Figure 3d cont.
BMS345541 + Rapamycin
Repeat 1 (used in densitometry)
P-STAT3 (Y705)
P-RpS6 (S235/236)
β-actin
STAT3
P-RelA (S536)

## Slide 12
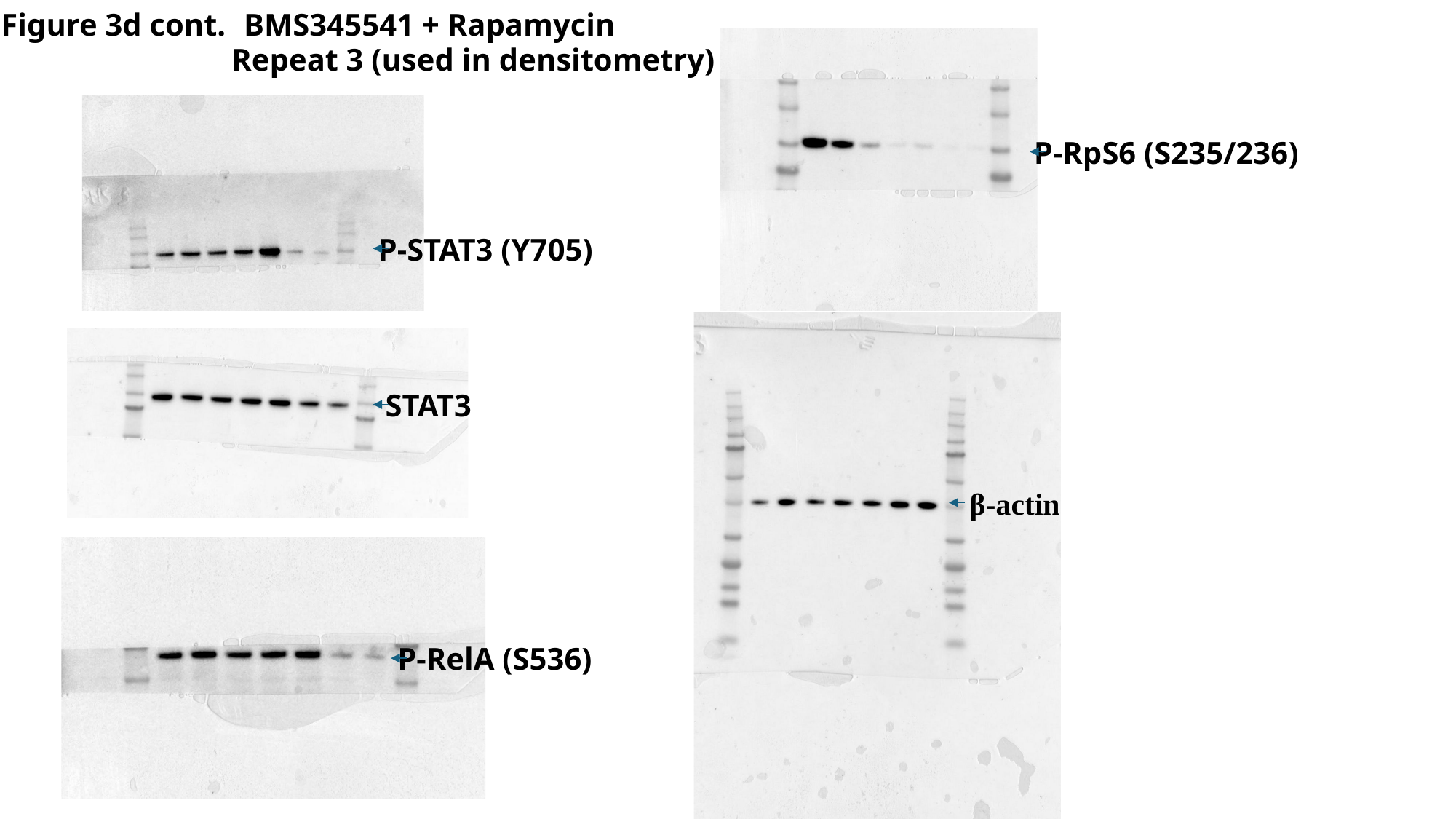

Figure 3d cont.
BMS345541 + Rapamycin
Repeat 3 (used in densitometry)
P-RpS6 (S235/236)
P-STAT3 (Y705)
STAT3
β-actin
P-RelA (S536)

## Slide 13
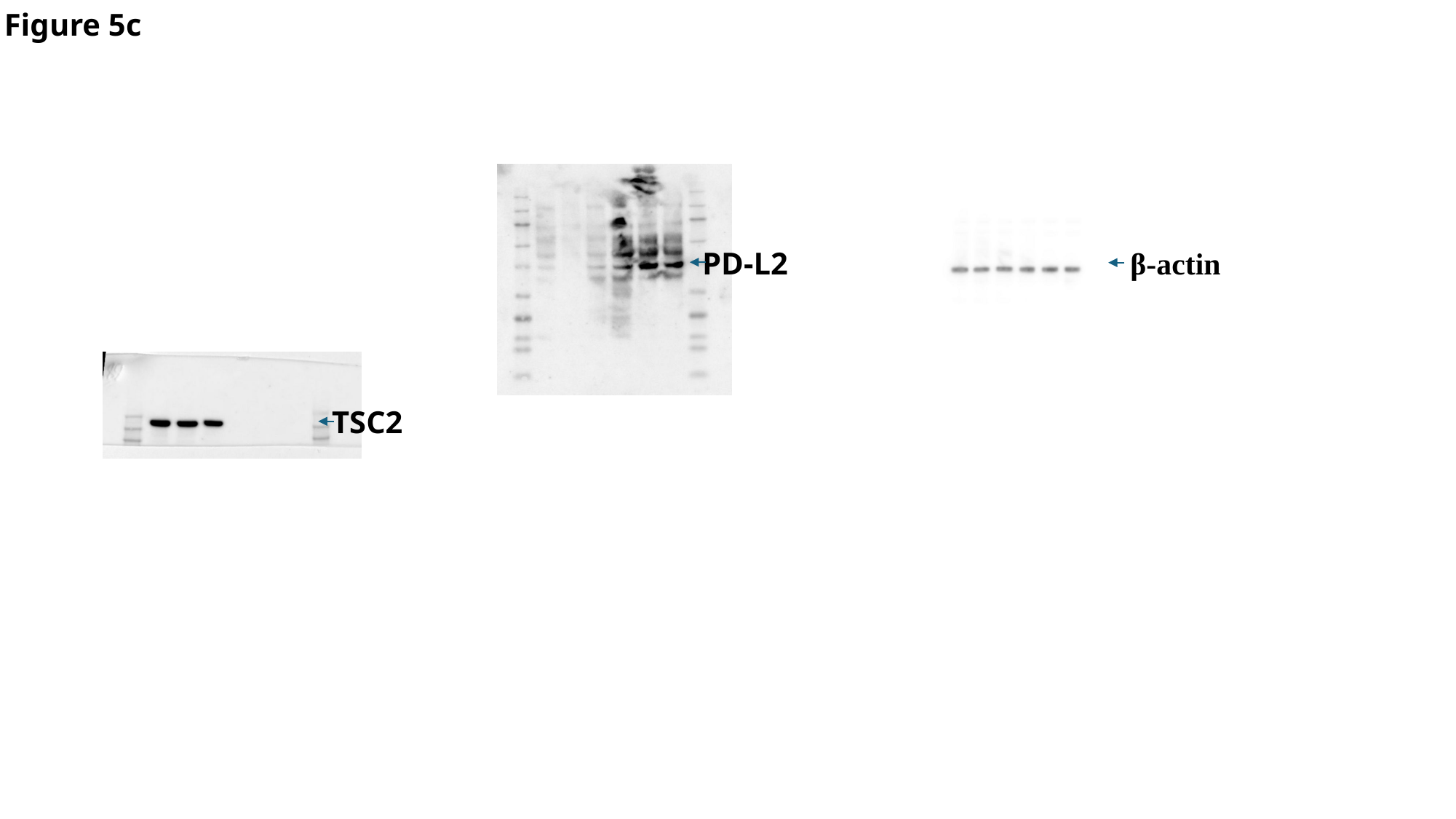

Figure 5c
PD-L2
β-actin
TSC2

## Slide 14
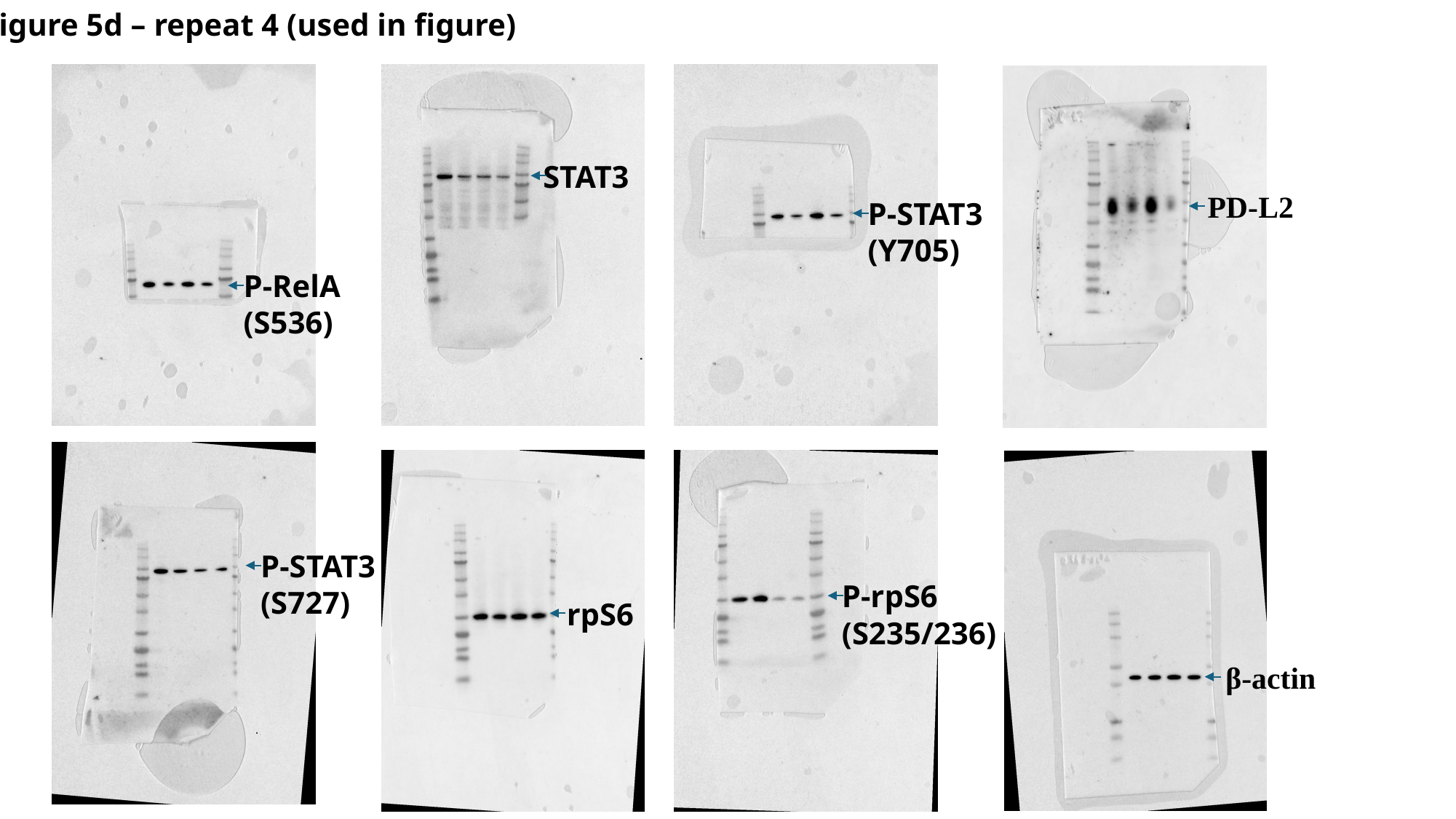

Figure 5d – repeat 4 (used in figure)
STAT3
PD-L2
P-STAT3
(Y705)
P-RelA
(S536)
P-STAT3
(S727)
P-rpS6
(S235/236)
rpS6
β-actin

## Slide 15
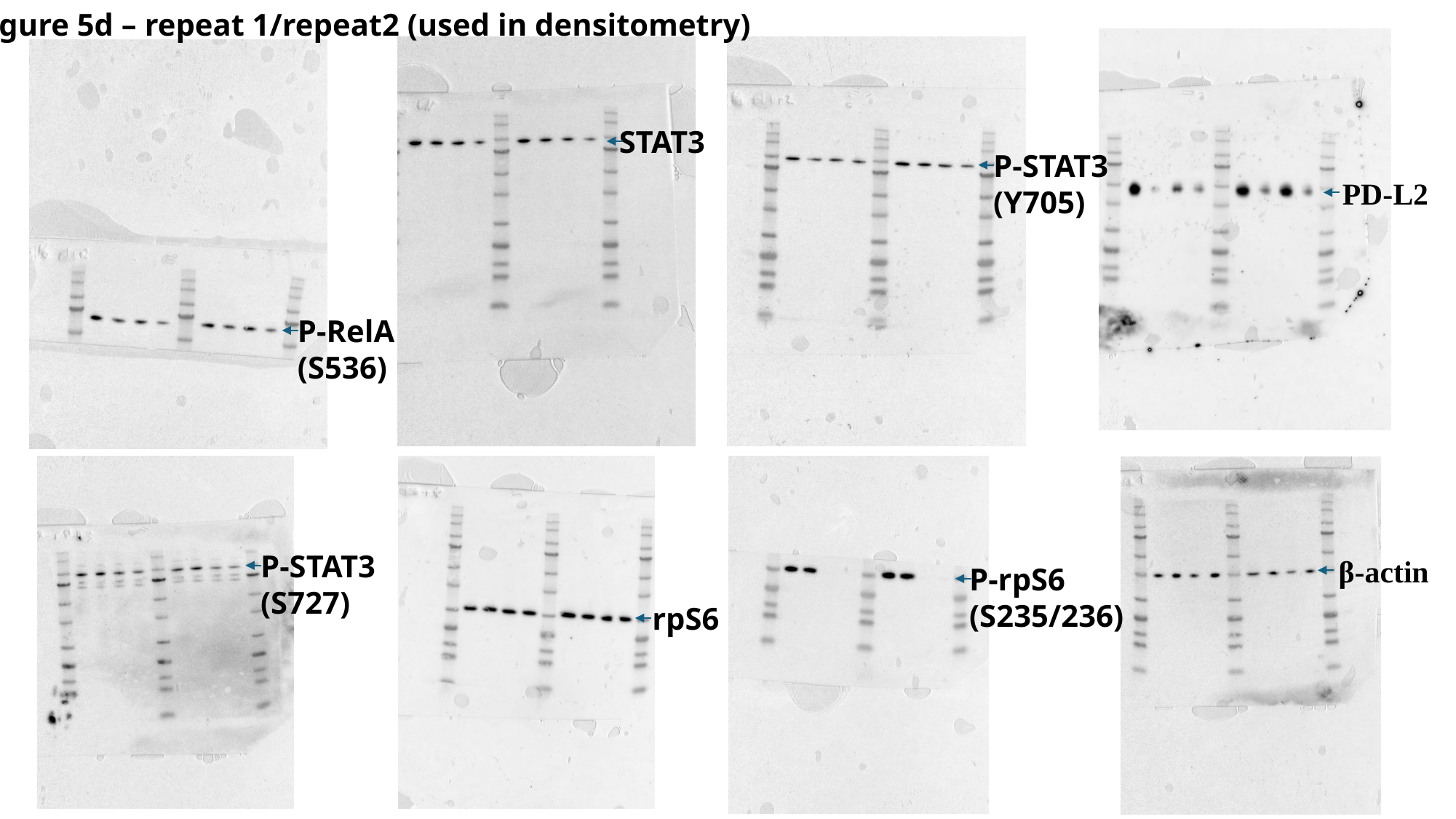

Figure 5d – repeat 1/repeat2 (used in densitometry)
STAT3
P-STAT3
(Y705)
PD-L2
P-RelA
(S536)
P-STAT3
(S727)
β-actin
P-rpS6
(S235/236)
rpS6

## Slide 16
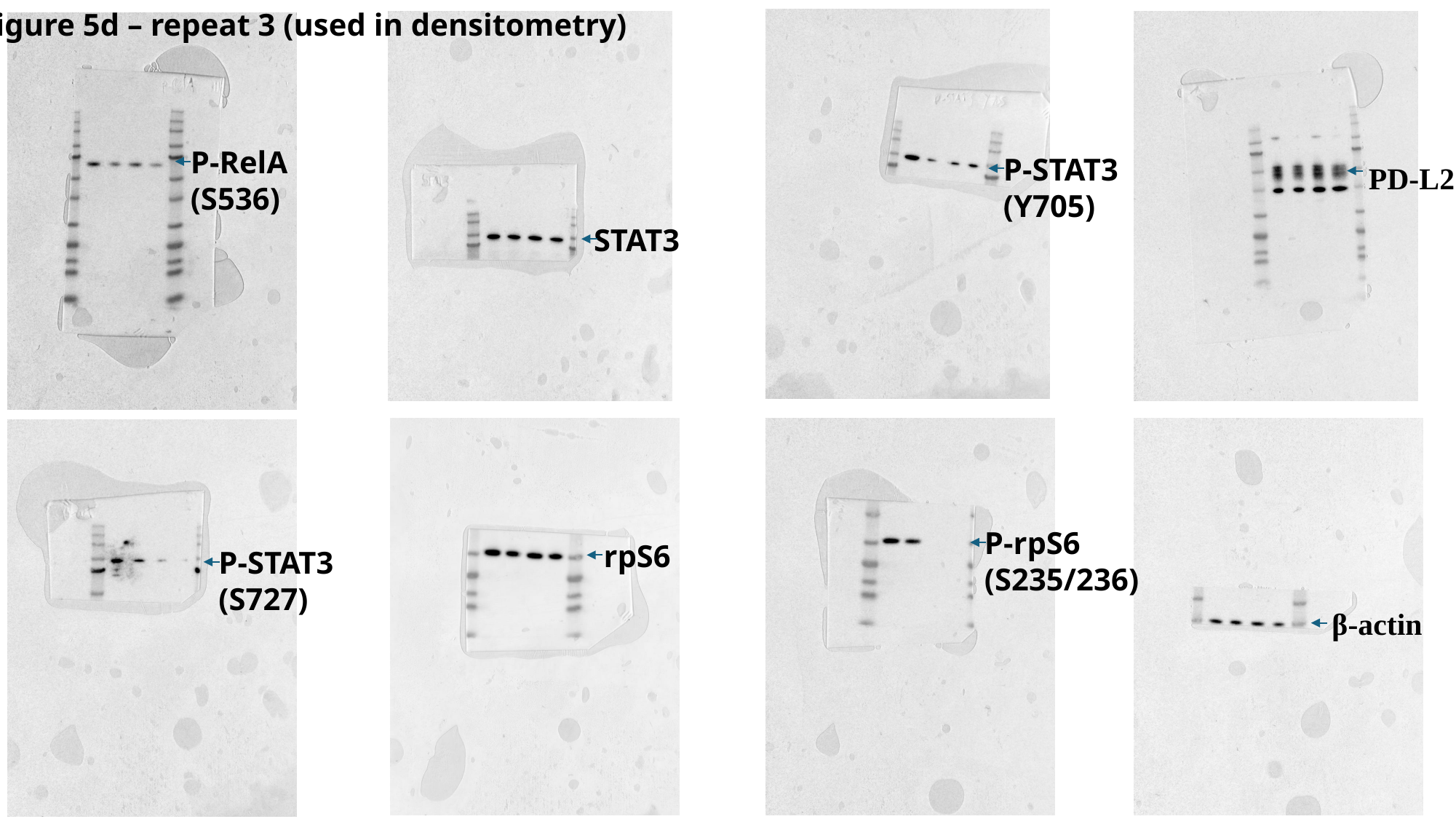

Figure 5d – repeat 3 (used in densitometry)
P-RelA
(S536)
P-STAT3
(Y705)
PD-L2
STAT3
P-rpS6
(S235/236)
rpS6
P-STAT3
(S727)
β-actin
