# Supplementary material for: Loss of tuberous sclerosis complex 2 confers inflammation via dysregulation of nuclear factor kappa-light-chain-enhancer of activated B cells
Source: J Inflamm (Lond). 2025 Sep 26;22:38. doi: 10.1186/s12950-025-00464-8 (PMC12465316; doi:10.1186/s12950-025-00464-8)
Supplement: Supplementary file 6 — Supplementary Material 6. [file 12950_2025_464_MOESM6_ESM.pptx]

## Slide 1
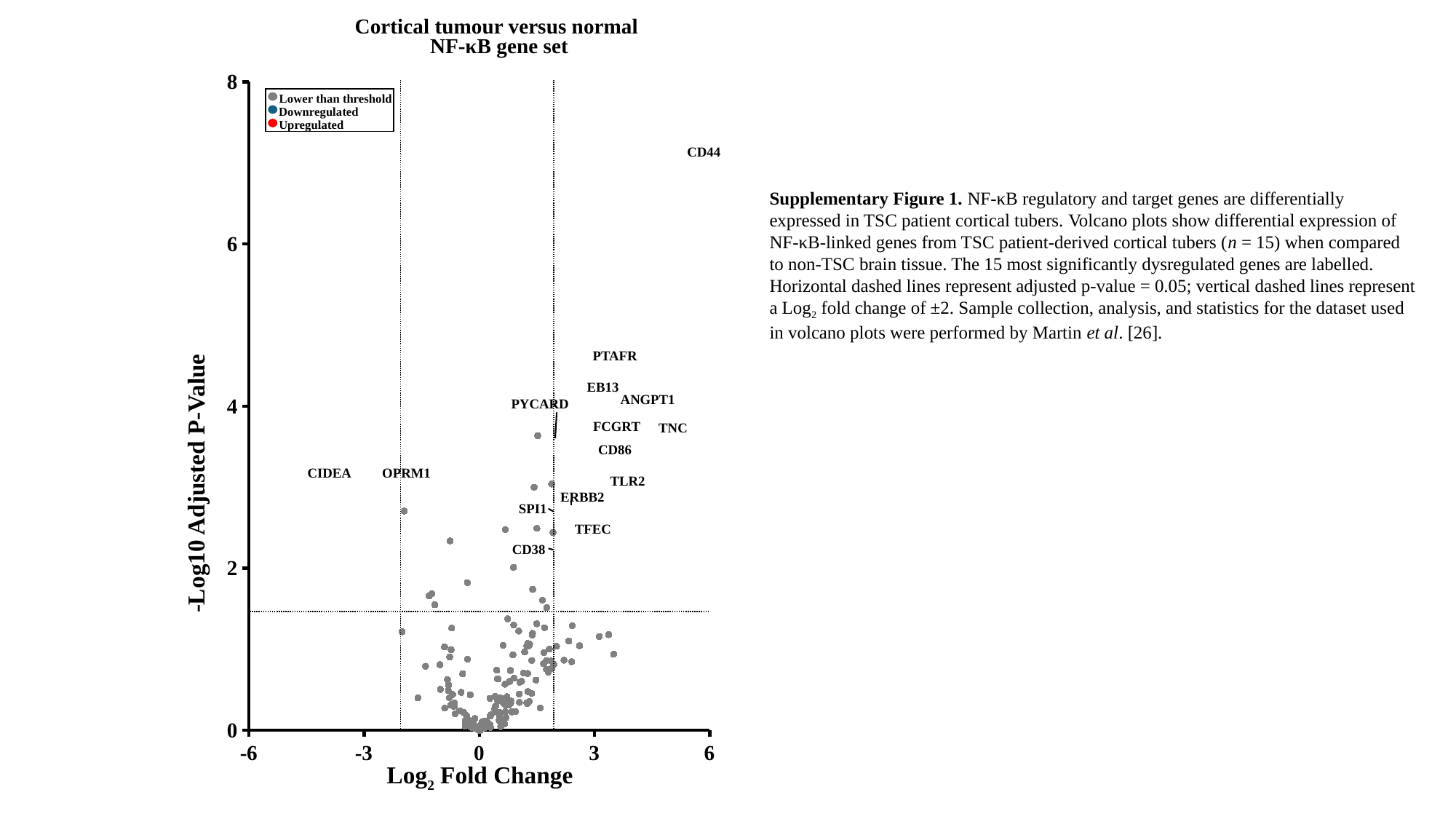

Cortical tumour versus normal NF-κB gene set
### Chart
| Category | | | |
|---|---|---|---|Lower than threshold
Downregulated
Upregulated
CD44
Supplementary Figure 1. NF-κB regulatory and target genes are differentially expressed in TSC patient cortical tubers. Volcano plots show differential expression of NF-κB-linked genes from TSC patient-derived cortical tubers (n = 15) when compared to non-TSC brain tissue. The 15 most significantly dysregulated genes are labelled. Horizontal dashed lines represent adjusted p-value = 0.05; vertical dashed lines represent a Log2 fold change of ±2. Sample collection, analysis, and statistics for the dataset used in volcano plots were performed by Martin et al. [26].
PTAFR
EB13
ANGPT1
PYCARD
FCGRT
TNC
CD86
CIDEA
OPRM1
TLR2
-Log10 Adjusted P-Value
ERBB2
SPI1
TFEC
CD38
Log2 Fold Change

## Slide 2
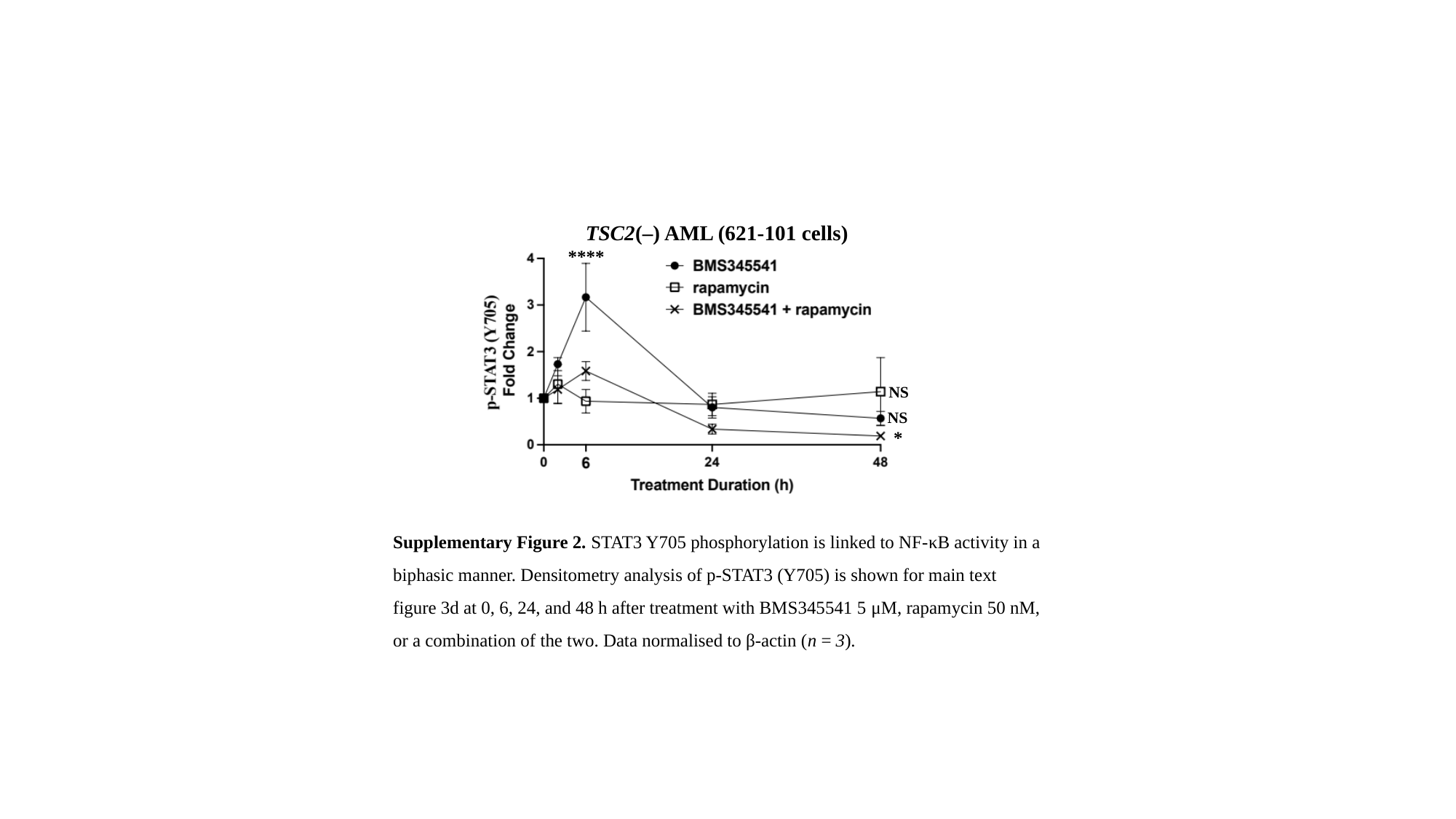

TSC2(–) AML (621-101 cells)
****
NS
NS
*
Supplementary Figure 2. STAT3 Y705 phosphorylation is linked to NF-κB activity in a biphasic manner. Densitometry analysis of p-STAT3 (Y705) is shown for main text figure 3d at 0, 6, 24, and 48 h after treatment with BMS345541 5 μM, rapamycin 50 nM, or a combination of the two. Data normalised to β-actin (n = 3).

## Slide 3
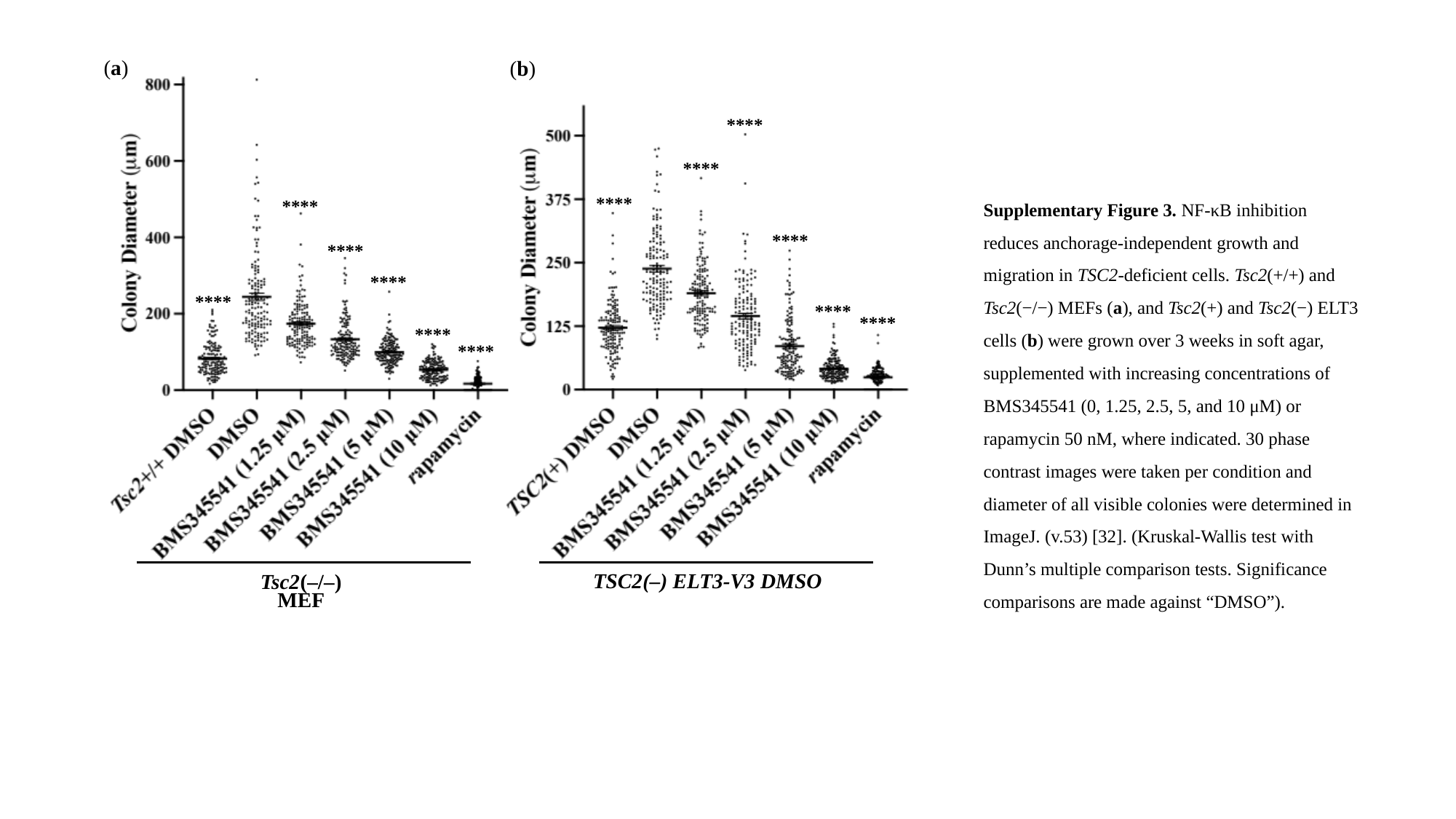

(a)
(b)
****
****
Supplementary Figure 3. NF-κB inhibition reduces anchorage-independent growth and migration in TSC2-deficient cells. Tsc2(+/+) and Tsc2(−/−) MEFs (a), and Tsc2(+) and Tsc2(−) ELT3 cells (b) were grown over 3 weeks in soft agar, supplemented with increasing concentrations of BMS345541 (0, 1.25, 2.5, 5, and 10 μM) or rapamycin 50 nM, where indicated. 30 phase contrast images were taken per condition and diameter of all visible colonies were determined in ImageJ. (v.53) [32]. (Kruskal-Wallis test with Dunn’s multiple comparison tests. Significance comparisons are made against “DMSO”).
****
****
****
****
****
****
****
****
****
****
TSC2(–) ELT3-V3 DMSO
Tsc2(–/–) MEF

## Slide 4
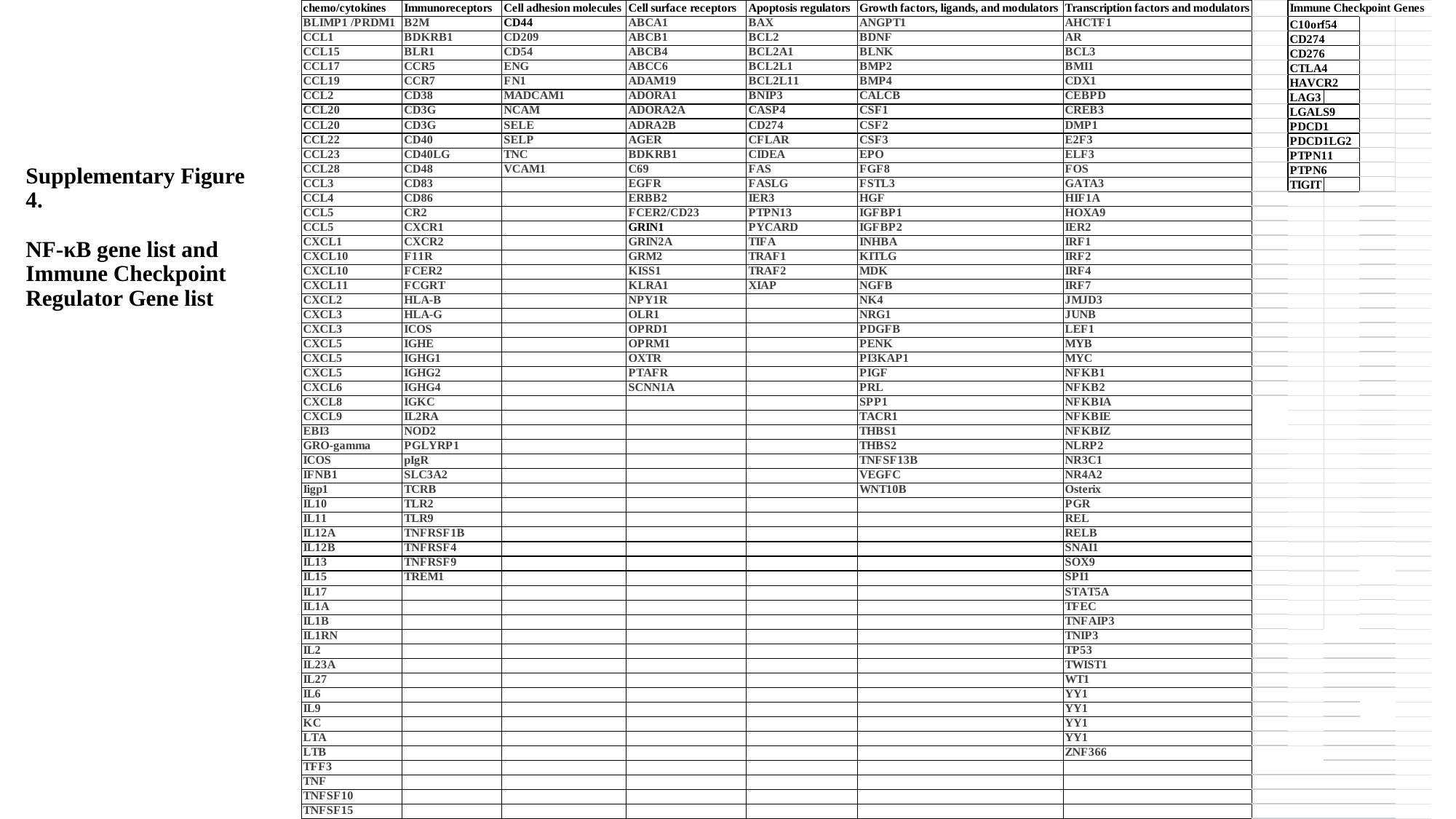

# Supplementary Figure 4. NF-κB gene list and Immune Checkpoint Regulator Gene list
